# DMSO solvates of *tert*-butyl­calix[6]arene and related multisolvent structures

**DOI:** 10.1107/S2052520625008625

**Published:** 2025-11-05

**Authors:** Monika Wanat, Ewelina Zaorska, Maura Malinska

**Affiliations:** a Faculty of Chemistry University of Warsaw Pasteura 1 Warsaw 02-093 Poland; Tianjin Normal University, People’s Republic of China

**Keywords:** calixarenes, solvates, crystallization, multicomponent crystal, intermolecular interactions, host–guest systems

## Abstract

*tert*-Butyl­calix[6]arene (TBC6) exhibits a rich crystal energy landscape, with structural diversity driven by macrocycle conformation, guest inclusion and self-assembly. Using multicomponent crystallization mixtures containing DMSO and additional solvents (*e.g*. DMF, chloro­benzene, 1,2-di­chloro­benzene, anisole, THF), we obtained seven new TBC6 solvates. One structure, crystallized in the presence of pyridine, adopts a winged-cone conformation with a bilayer arrangement in the crystal. The remaining six are isostructural and feature a columnar packing of TBC6 in a 1,2,3-alternate conformation stabilized by four strong interactions with DMSO: two hydrogen bonds and two C—H⋯π interactions. Notably, DMSO in the TBC6 cavity is released at elevated temperatures (∼200°C), consistent with its stronger binding energy as calculated using DFT methods. TBC6–TBC6 interactions, both within and between columns, dominate the crystal packing, with intracolumnar contacts contributing twice as much stabilization as intercolumnar ones, where additional solvent guests are located. Hirshfeld surface analysis supports these findings, highlighting extensive contact areas between TBC6 molecules. This study contributes to understanding how solvent properties, interaction energies, and packing preferences drive the solid-state organization of calixarenes—molecules with broad applications in separation, recognition, and supramolecular chemistry.

## Abstract 2

This study highlights how solvent properties, host–guest interaction energies and packing preferences govern the solid-state organization of *tert*-butyl­calix[6]arene, a macrocycle of importance in separation, molecular recognition and supramolecular chemistry.

## 1. Introduction

Calix[6]arenes are macrocyclic compounds composed of six phenolic units interconnected via methyl­ene bridges at the *ortho* positions (C-2 and C-6). Their unique structural features, particularly their conformational flexibility, enable them to engage in non-covalent interactions with guest molecules (Baldini *et al.*, 2020 ▸; de Fátima *et al.*, 2009 ▸; Muley *et al.*, 2024 ▸; Wu *et al.*, 2024 ▸; Nag & Rao, 2022 ▸). These interactions extend beyond small molecules to include proteins (Flood *et al.*, 2024 ▸; Alex *et al.*, 2019 ▸; Flood *et al.*, 2022 ▸), making calix[6]arenes valuable models for studying non-covalent interactions in various systems. (Amezcua *et al.*, 2024 ▸; Hurley *et al.*, 2023 ▸).

Beyond fundamental research applications, calix[6]arenes show significant potential in practical uses. Their water-soluble derivatives exhibit low cytotoxicity and immunogenicity, making them suitable for drug delivery systems, photodynamic therapy, and enzyme mimicry (Razuvayeva *et al.*, 2020 ▸; Paclet *et al.*, 2006 ▸; Talukdar *et al.*, 2023 ▸; Cvetnić *et al.*, 2024 ▸). Specific examples of their applications include their use as stationary phases in capillary gas chromatography (*e.g.tert*-butyl­(tetra­decyl­oxy)calix[6]arene) (Sun *et al.*, 2019 ▸), selective extraction agents for bioactive molecules from natural products (*e.g.* 4-*tert*-butyl-calix[6]arene) (Segneanu *et al.*, 2016 ▸) and as active ingredients in emulsions for treating uranium skin contamination (1,3,5-OCH_3_-2,4,6-OCH_2_COOH-*p*-*tert*-butyl­calix[6]arene) (Phan *et al.*, 2013 ▸).

**Figure 1:**
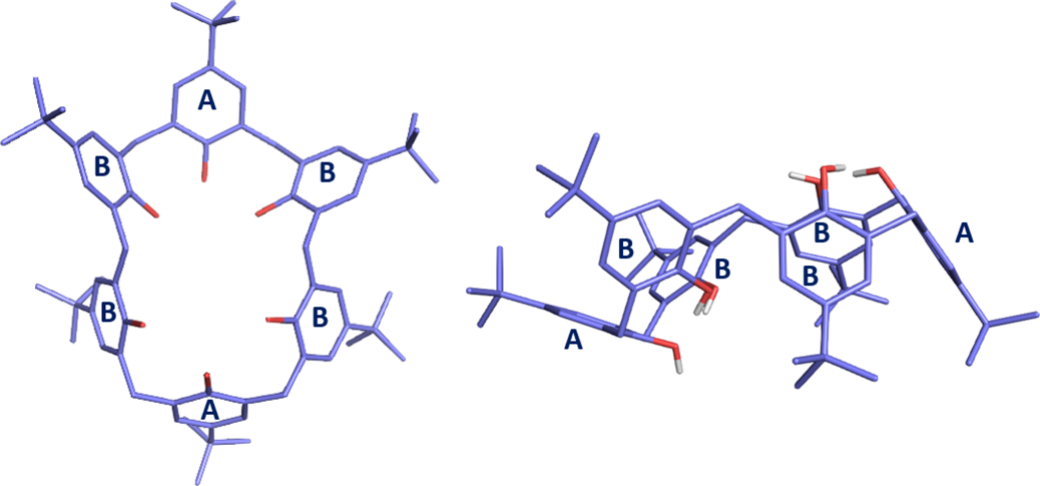
Structure of TBC6 in the 1,2,3-alternate conformation, viewed from the top (left) and the side (right). Aromatic rings are designated A-type and B-type. Hydrogen atoms are omitted for clarity.

The conformational diversity of calix[6]arenes further enhances their versatility. Molins *et al.* (1992 ▸) characterized the winged-cone conformation, where four benzene rings orient perpendicular to the molecular base while two rings at the 1,4-positions remain flat. In contrast, the 1,2,3-alternate conformation features three hydroxyl groups on the upper rim and three on the lower rim (Fig. 1 ▸). Low-temperature solution NMR spectroscopy identifies the winged-cone as the predominant conformation (Martins *et al.*, 2017 ▸), with energy differences of 50 kJ mol^−1^ (winged-cone) and 66 kJ mol^−1^ (1,2,3-alternate) relative to the pinched cone conformation of *p*-*tert*-butyl­calix[6]arene (TBC6, Fig. 1 ▸). (Wolfgong *et al.*, 1996 ▸). The preferred conformation depends on *para*-position substituents: sulfate derivatives favor the 1,2,3-alternate form, while bulky groups (*e.g.* benzyl, *tert*-butyl) stabilize the pinched cone.

Crystallographic studies provide further insights into these conformations. While calix[6]arenes typically adopt the pinched-cone conformation in the solid state, TBC6 forms a 1,2,3-alternate conformation when crystallized with di­methyl sulfoxide (DMSO) (Suwinska, 2016 ▸). Recent studies have identified new winged-cone structures with solvents including DMSO, di­methyl­formamide (DMF) and pyridine (Martins *et al.*, 2017 ▸; Malinska, 2021 ▸), which commonly form layered TBC6–solvent arrangements.

Typically, TBC6 solvates crystallize in monoclinic space group *P*2_1_/*c*, with one guest molecule occupying the TBC6 cavity (interaction energy of approximately −50 kJ mol^−1^). The apolar surface area to volume ratio (aPSA/*V*) serves as a useful metric for distinguishing between exclusion and inclusion complexes, with a threshold value of 40. Solvent size significantly influences crystal packing, where small molecules (< 100 Å^3^) promote offset bilayer packing through strong TBC6 dimers (interaction energies < −110 kJ mol^−1^), while larger molecules prevent bilayer formation (Malinska, 2022 ▸).

TBC6 forms isostructural 1:1 complexes with various solvents (cyclo­hexane, benzene, toluene *etc*.), while different stoichiometries emerge at higher temperatures (1:3 for benzene/pyridine/DCM) or with small guests (1:2–1:4 for DCM; 1:2–2:3 for aceto­nitrile). Notably, TBC6 selectively incorporates chloro­benzene from 12 solvent mixtures (Zaorska *et al.*, 2023 ▸), demonstrating potential for solvent purification. This work extends these findings through systematic analysis of TBC6–DMSO–solvate crystal structures.

## 2. Experimental

### 2.1. Crystallization

TBC6 was purchased from Sigma Aldrich (USA). All solvents used for crystallization were purchased from POCH SA (Poland). All chemicals purchased were reagent grade and were used without further purification. For crystallization experiments, TBC6 was dissolved in mixtures of miscible solvents, prepared with equal volumes of each component, resulting in a single-phase system. Water was not considered as a potential component, since TBC6 is insoluble in water. In all cases, samples containing DMSO were handled in closed vials to avoid moisture uptake; under these conditions we have never observed precipitation of TBC6. Samples of TBC6 were dissolved with solvent mixtures that contain DMSO at values of temperature in range 25°C to 60°C. Heated samples were slowly cooled down and all samples were left for slow evaporation in an incubator at 24.5°C. Below details are given of the crystallization experiments that lead to obtaining crystals **1**–**7**.

#### 2.1.1. 2TBC6·2DMSO·8DMSO (**1**)

TBC6 (20.8 mg) was dissolved in a mixture of DMSO, benzene, di­chloro­methane (DCM) and pyridine (0.60 ml each) at room temperature. Crystals of **1** formed after a week.

#### 2.1.2. TBC6·2DMSO·DMF (**2**)

TBC6 (10.5 mg) was dissolved in a solvent mixture containing DMSO, ethyl acetate, DMF and pyridine (0.20 ml of each) at room temperature. Crystals of **2** formed after a week.

#### 2.1.3. TBC6·2DMSO·0.66DMSO·0.34anisole (**3**)

TBC6 (3.5 mg) was dissolved in a solvent mixture containing DMSO, anisole and *n*-heptane (0.35 ml of each) at 60°C. The crystallization sample was then slowly cooled to room temperature and the solution was left for slow evaporation which resulted in the crystallization of the desired compound. Crystals of **3** formed after 15 days.

#### 2.1.4. TBC6·2DMSO·0.63chlorobenzene·0.37benzene (**4**)

TBC6 (4.7 mg) was dissolved in a solvent mixture containing DMSO, benzene, pyridine, DCM, cyclo­hexane, toluene, methyl acetate, THF, chloro­benzene, anisole, *n*-heptane and MeCN (0.1 ml of each) at room temperature. Crystals of **4** formed after a week.

#### 2.1.5. TBC6·3DMSO (**5**)

TBC6 (10.0 mg) was dissolved in a solvent mixture containing DMSO and ethanol (0.40 ml of each) at 40°C. The crystallization sample was then slowly cooled to room temperature and the solution was left for slow evaporation (at room temperature, incubator, 24.5°C) which resulted in crystallization of the final compound. The first crystals of **5** formed after two weeks.

#### 2.1.6. TBC6·2DMSO·0.41(1,3-dichlorobenzene)·0.59THF (**6**)

TBC6 (10 mg) was dissolved in a solvent mixture containing 1,2-di­chloro­benzene, 1,3-di­chloro­benzene, 1,4-di­chloro­benzene (0.07 ml of each) and DMSO, benzene, pyridine, DCM, cyclo­hexane, toluene, methyl acetate, THF, chloro­benzene, anisole, *n*-heptane, MeCN, ethyl acetate (0.20 ml of each) at room temperature. Crystals of **6** formed after six days.

#### 2.1.7. 2TBC6·4DMSO·2(1,2-dichlorobenzene) (**7**)

TBC6 (10 mg) was dissolved in a solvent mixture containing DMSO and 1,2-di­chloro­benzene (0.2 ml of each) at room temperature. Crystals of **7** formed after 11 days.

### 2.2. Single-crystal X-ray diffraction measurements

Colorless transparent well formed complex crystals were used to collect X-ray diffraction data on an Agilent SuperNova diffractometer controlled by *CrysAlis PRO* (Rigaku, 2015 ▸) software and equipped with a Cu *K*α micro-focus X-ray source (λ = 1.54 Å, 50.0 kV, 0.8 mA) and a HyPix detector. Experiments were carried out at 100.0 (2) K using an Oxford Cryosystems cooling device. Crystals were mounted on a MiTeGen mount with a droplet of immersion oil and immediately cooled. Each crystal was positioned 54.0 mm from the detector. Indexing and integration were performed with *CrysAlis PRO* software. Selected crystallographic data are found in Table 1 ▸ and full data are in Table S1.

### 2.3. Structure solution and refinement

Structures were solved using *SHELXT* (Sheldrick, 2015 ▸) and refined using *SHELXL* (Sheldrick, 2015 ▸) in *Olex2* (Dolomanov *et al.*, 2009 ▸) software. Refinement was based on *F*^2^ for all reflections except those with negative intensities. Weighted *R* factors (*wR*) and all goodness-of-fit values (*S*) were based on *F*^2^, whereas conventional *R* factors were based on amplitudes, with *F* set to zero for negative *F*^2^. Atomic scattering factors were taken from *International Tables for Crystallography* (Prince & Spiegelman, 1992 ▸).

Disorders were found in the structures **1**–**4** and **6**–**7**. Disorder was refined in these structures. If necessary, restraints on bonds (**1**–**4**, **6**, **7**), thermal parameters (**3**) as well as constraints on thermal parameters (**1**–**4**, **6**) were applied. Mostly, rotational disorder occurs within the *tert*-butyl groups without affecting the quaternary carbons. However, if the more than one *tert*-butyl group is disordered, then the occupancies between those groups may be different. These occupancies are as follows: **1** 0.50/0.50, **2** 0.57/0.43 and 0.58/0.42,**3** 0.66/0.34, **4** 0.54/0.46, 0.73/0.27 and 0.85/0.15, **6** 0.70/0.30 and **7** 0.78/0.22. Disorder of the solvents was found in the structures **1**, **2**, **3**, **4** and **6**. Rotational disorder occurs for the DMSO molecule in structure **1** (0.62/0.38) and DMF solvent in structure **2** (0.75/0.25). Substitutional disorder occurs for structures **3**, **4** and **6**, with a different solvent at the same position *i.e.***3** DMSO and anisole (0.66/0.34), **4** chloro­benzene and benzene (0.63/0.37) and **6** THF and 1,2-di­chloro­benzene (0.59/0.41).

In the structure **1**, diffuse electron densities were found in the difference Fourier maps and are ascribable to nonstoichiometric solvent molecule charge densities. However, even after several trial refinements, attempts to assign these weak residual electron-density peaks to low-occupancy atomic sites were unsuccessful, and the data were processed with *PLATON SQUEEZE* (Spek, 2015 ▸) prior to the final refinement. A solvent mask was calculated and 343 electrons were found in a volume of 864 Å^3^ in one void per unit cell. This is consistent with the presence of eight C_2_H_6_SO per asymmetric unit, which corresponds to 336 electrons per unit cell.

### 2.4. Theoretical calculations

Bond lengths involving H atoms were normalized to mean neutron values using the *LSDB* software (Jarzembska *et al.*, 2017 ▸). In the case of disordered structures, calculations were performed using the atomic positions corresponding to the major-occupancy sites.

Energy framework calculations were carried out with *CrystalExplorer 3.3* (Turner *et al.*, 2014 ▸), using the Hartree–Fock method with the 3-21G basis set. Frameworks were visualized along the *a*, *b* and *c* axes with a scale factor of 50 and an energy threshold of 5 kJ mol^−1^ (Table S7).

Counterpoise-corrected interaction energies were calculated with *Gaussian16* (Frisch *et al.*, 2016 ▸) for selected dimers extracted from the crystal structures. These calculations employed density functional theory with the B3LYP functional and the 6-31G(d,p)_6d_10f basis set, including dispersion corrections (Grimme *et al.*, 2010 ▸). The basis set superposition error was accounted for by applying the standard counterpoise correction (Boys & Bernardi, 1970 ▸; Simon *et al.*, 1996 ▸).

At the same level of theory, three representative conformations of TBC6 (low-energy pinched cone, winged-cone and 1,2,3-alternate) were extracted from crystal structures. Geometry optimizations were performed to assess the relative conformational energies.

### 2.5. Thermogravimetric analysis

To confirm the presence of solvent mixtures in the selected structures, *i.e.***2** and **5** thermogravimetric analyses (DSC-TGA) were performed using a Mettler-Toledo Star. Al_2_O_3_ was used as the reference material, and the heating and cooling rates were 5 K min^−1^ with an air flux of 90 cm^3^ min^−1^. The data are presented in Fig. S1. DSC–TGA measurements were performed only for selected structures, because crystals of several solvates were too large and required grinding prior to analysis, which induced desolvation/recrystallization and produced phases different from the single-crystal forms (to be reported separately). Composition for these samples was determined by single-crystal X-ray diffraction modelling. For each sample, at least 20 single crystals were examined and yielded consistent unit-cell parameters (within experimental uncertainty), indicating that the single-crystal composition is representative of the bulk. Visual inspection of the bulk material indicated homogeneous batches, with crystals of similar size and morphology.

## 3. Results and discussion

### 3.1. TBC6 solvate structures

**Figure 2:**
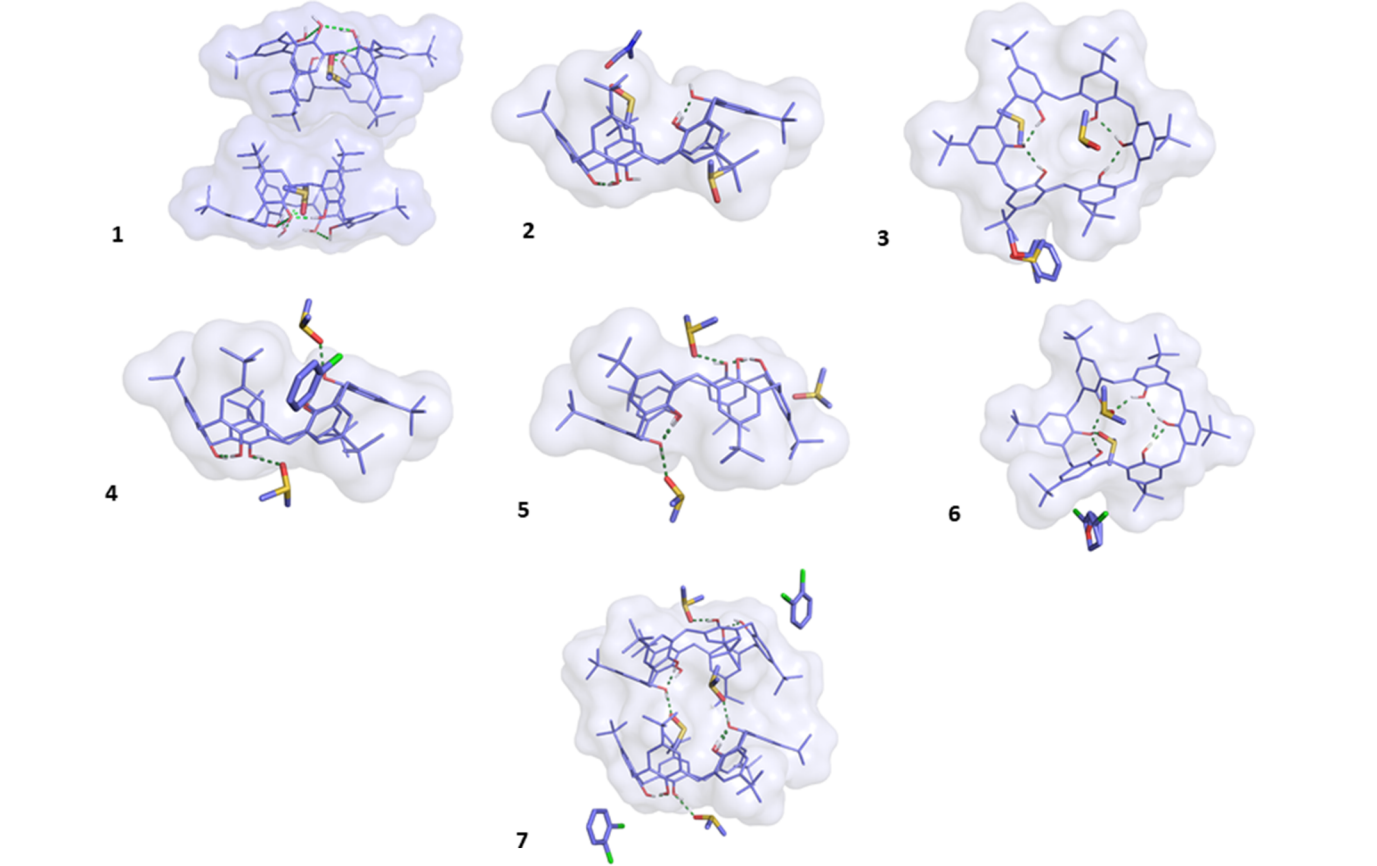
Asymmetric units of **1**–**7** shown from selected views (either from the top or from the side). Hydrogen atoms bonded to carbon (non-polar hydrogens) are omitted for clarity. TBC6 is shown as solid lines with a semi-transparent van der Waals surface, and solvent molecules are shown as sticks. Hydrogen bonds are shown as green dashed lines. Views from the top and the side for all structures are provided in Fig. S2.

Seven TBC6 solvate structures (**1**–**7**) were obtained from crystallization experiments using various solvent mixtures and characterized by single-crystal X-ray diffraction. The asymmetric units are shown in Fig. 2 ▸.

Structure **1** belongs to the family of layered TBC6 structures, similar to the previously reported DMSO solvate REGWEI (Martins *et al.*, 2017 ▸) (Table S3) and pyridine solvates (Malinska, 2021 ▸). It adopts a double-layer arrangement built from TBC6 molecules in the winged-cone conformation, each containing one DMSO molecule in the cavity. The structure crystallizes in triclinic space group *P*1 with two TBC6 molecules in the asymmetric unit, each forming hydrogen bonds with an included DMSO molecule. The O atom of DMSO accepts two hydrogen bonds from opposite hydroxyl groups of TBC6. In this conformation, the phenyl rings lie relatively flat, enabling closer approach of the hydroxyl groups. Within each layer, every TBC6 molecule is surrounded by six neighbors via C—H⋯π interactions. Adjacent layers interact through dispersive contacts between *tert*-butyl groups, resulting in a shift of approximately half the TBC6 length; the *tert*-butyl groups act as lids closing the TBC6 binding pockets. Additional disordered solvent molecules form alternating layers, which could not be modeled explicitly and were treated using the solvent-masking procedure.

The key structural difference between REGWEI and structure **1** lies in the relative orientation of the double layers. In REGWEI, *tert*-butyl groups of neighboring TBC6 molecules are positioned face-to-face, giving an interlayer spacing (averaged over six O atoms) of 15.31 Å. In contrast, in structure **1**, the *tert*-butyl groups of one layer interdigitate with those of the next, resulting in a shorter interlayer distance of 14.17 Å. Thus, structure **1** represents a more compact layered packing compared to REGWEI.

Structures **2**–**7** exhibit a 1:3 TBC6–guest ratio. In all of these, TBC6 adopts a 1,2,3-alternate conformation stabilized by four intramolecular and two intermolecular hydrogen bonds involving DMSO molecules (Tables S10–S16). Although the 1,2,3-alternate and winged-cone conformations (Fig. 2 ▸) recalculated to be higher in energy than the pinched-cone form (by *ca* 52.7 and 49.9 kJ mol^−1^, respectively), both appear in the solid state, stabilized by favorable TBC6–guest and TBC6–TBC6 interactions. This demonstrates that crystal packing stabilization can overcome intrinsic conformational preferences.

In all structures **2**–**7**, at least two DMSO molecules per TBC6 unit are present (Fig. 2 ▸). The 1,2,3-alternate conformation is defined by the alternating orientation of hydroxyl groups: three above and three below the mean macrocyclic plane. This conformational change results from the disruption of two of the six intramolecular hydrogen bonds that stabilize the lowest-energy, pinched-cone conformation. In this alternate arrangement, the same hydroxyl groups form four intramolecular hydrogen bonds across all structures in the series.

Each of these crystal structures contains three guest solvent molecules per TBC6: two DMSO molecules and one additional solvent of a different type. While minor geometric variations are observed among them, the conformation of TBC6 is well preserved. These differences primarily arise from variations in the size and nature of the guest molecules (Table S2).

**Figure 3:**
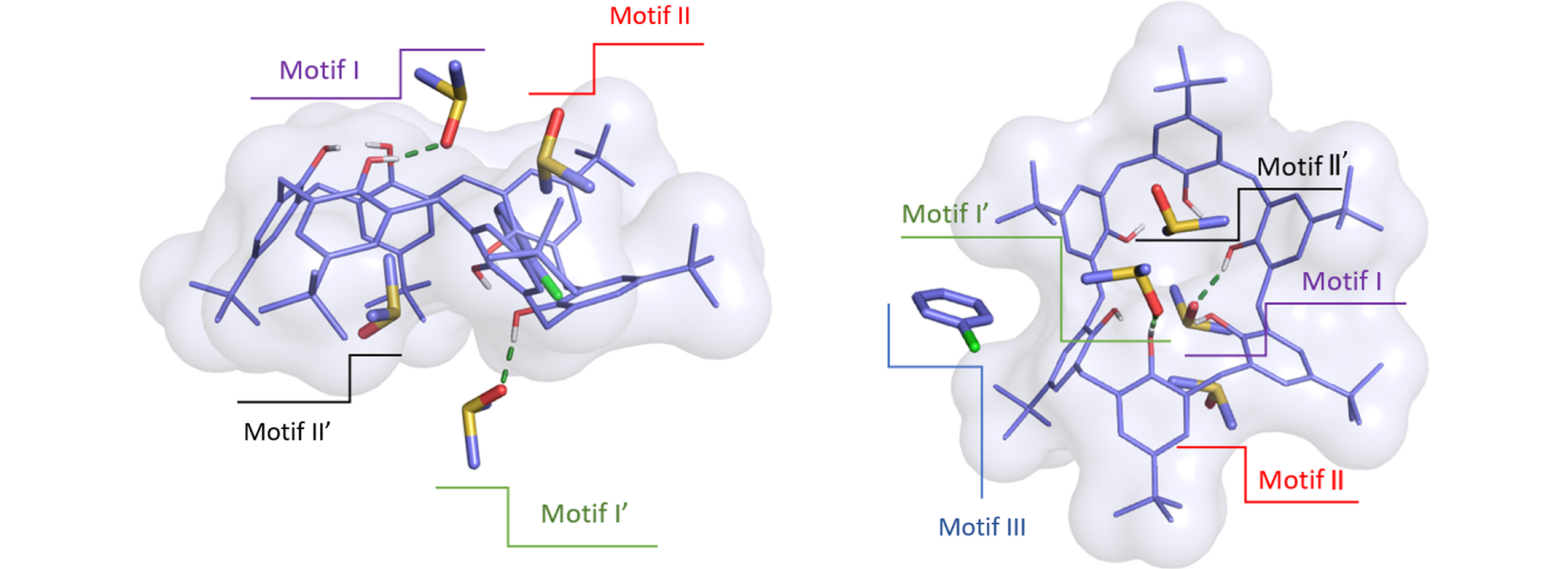
TBC6–solvent interaction motifs in presented structures, based on the example of structure **4**. Motifs I, I′, II and II′ are intracolumnar guests, whereas motif III is intercolumnar guest. Motifs I and I′ are hydrogen bonds, whereas motifs II, II′, and III are C—H⋯π interactions.

In addition, each TBC6 molecule interacts with four DMSO molecules located above and below it, forming hydrogen bonding and C—H⋯π-interaction motifs labeled I, I′, II and II′, as shown in Fig. 3 ▸. Motifs I and I′ differ in the hydroxyl group acting as hydrogen-bond acceptor: motif I involves a terminal hydroxyl group from one triad, engaged in a single intramolecular hydrogen bond; motif I′ involves a central hydroxyl group from the opposite triad, accepting two intramolecular hydrogen bonds.

**Figure 4:**
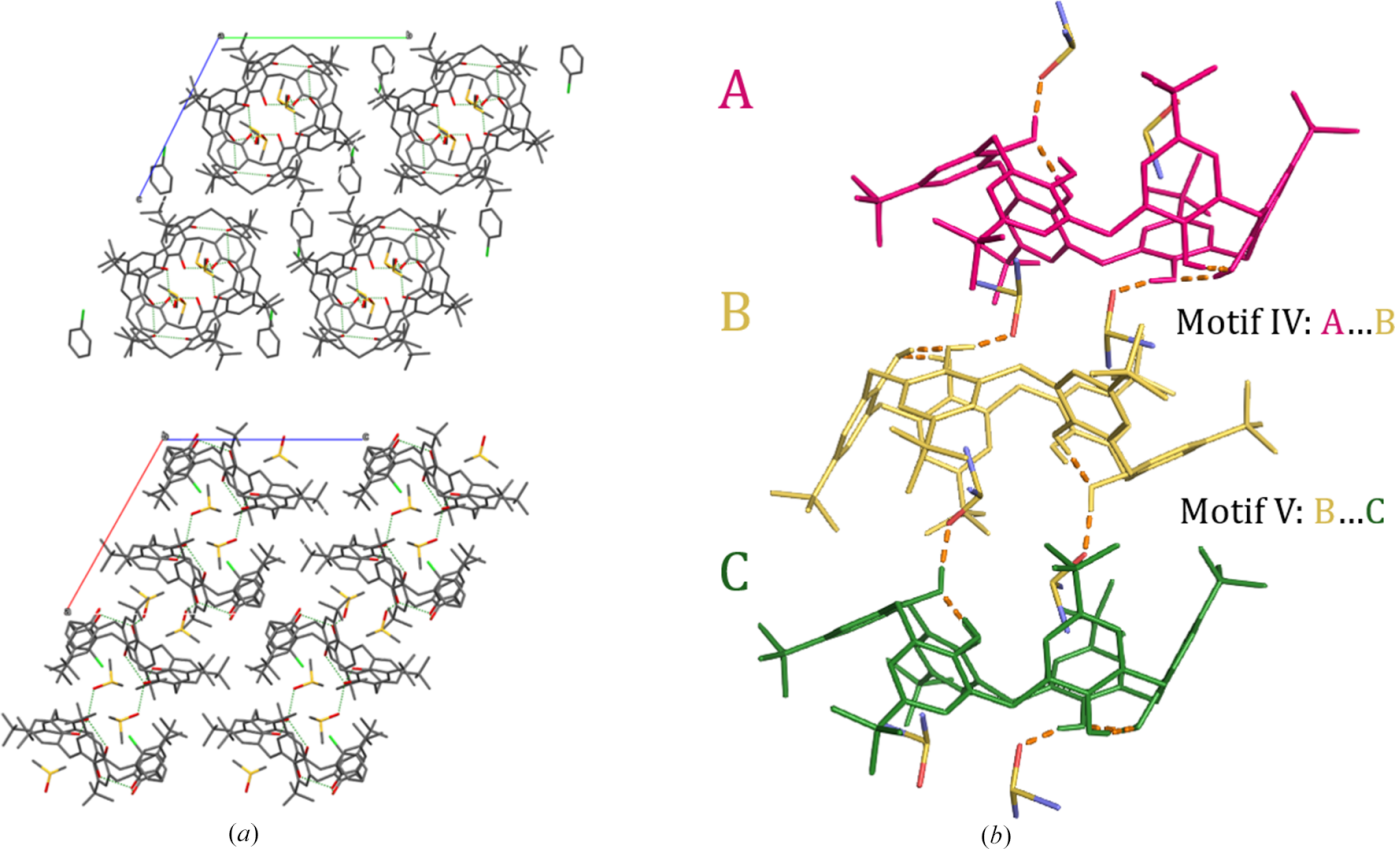
(*a*) Crystal packing of **4** viewed along *a* (top) and along *b* (bottom). The view along *a* shows four columns; the view along *b* shows two columns in side profile. Columns comprise TBC6 and DMSO; chlorobenzene molecules occupy intercolumnar sites. Hydrogen bonds are shown as green dashed lines. Top and side views of a column for all structures are provided in Table S3. (*b*) TBC6–TBC6 motifs IV and V within a single column. H atoms are omitted for clarity; hydrogen bonds are shown as orange dashed lines. A, B and C denote symmetry-related TBC6 molecules.

The symmetry-equivalent DMSO molecule involved in motif I occupies the cavity of a neighboring TBC6 molecule and forms a network of C—H⋯π interactions, where the methyl groups of DMSO act as hydrogen donors. This is designated as motif II. Similarly, the DMSO involved in motif I′ fills the remaining void and forms additional C—H⋯π interactions with TBC6, referred to as motif II′. These interactions help to extend the structure into extended columns; a feature common to all structures **2**–**7** (Fig. 4 ▸).

The intercolumnar solvent molecule provides minor structural diversity. In structures **2**, **5** and **7** there are single solvent molecules: DMF in structure **2**, DMSO in structure **5** and 1,2-di­chloro­benzene in structure **7**. In contrast, structures **3**, **4** and **6** contain two solvent molecules in substitutional disorder, with their occupancies summing to one. Specifically, in structure **3**, the disordered site is occupied by DMSO and anisole, with occupancies of 0.66 and 0.34, respectively. In structure **4**, the intercolumnar guests are chloro­benzene and benzene, with occupancies of 0.63 and 0.37, respectively. Structure **6** contains THF and 1,3-di­chloro­benzene as the disordered guests, with occupancies of 0.59 and 0.41, respectively. These lateral interactions between TBC6 and the intercolumnar solvent molecules will be referred to as motif III. Across all structures, the solvent molecules fill between 22.1% and 31.3% of the unit-cell volume, as summarized in Table S9.

The extended columns are formed through alternating interactions between TBC6 and DMSO molecules, demonstrating that this packing motif is consistently favored regardless of the identity of the additional solvent used during crystallization. These other solvents simply occupy the space between the columns (Figs. 4 ▸ and 5 ▸, and Table S3). Each column consists of intracolumnar DMSO molecules that connect TBC6 molecules through a combination of hydrogen bonds and C—H⋯π interactions. Because the DMSO molecules fit snugly into the cavity of TBC6, the macrocycles still can engage in C—H⋯π interactions, where the donor C—H groups originate from the *tert*-butyl substituents. Since each TBC6 molecule interacts with different DMSO molecules above and below, two distinct types of interactions between neighboring TBC6 molecules within the columns can be distinguished—referred to as motifs IV and V [Fig. 4 ▸(*b*)]. Both involve C—H⋯π contacts, but differ in geometry due to variations in the binding modes of the DMSO molecules and their involvement in hydrogen bonding.

**Figure 5:**
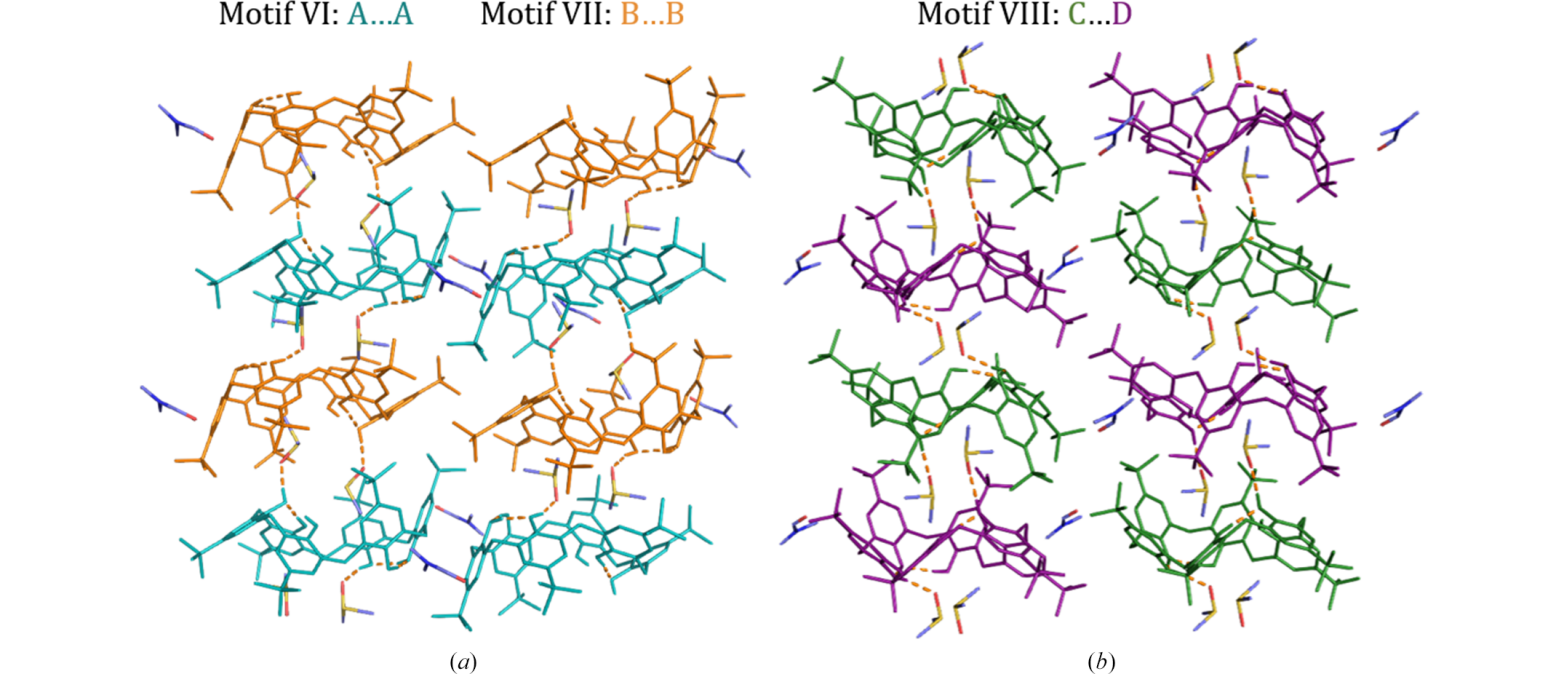
TBC6–TBC6 interactions between the columns: motifs (*a*) VI, VII, and (*b*) VIII, based on the example of structure **2**.

The TBC6 molecules also interact with neighboring columns through a network of weak dispersive forces (Table S6). Due to the elliptical shape of the TBC6 macrocycle—with one side shorter and the other longer—two distinct modes of intercolumnar interaction are observed. The shorter sides engage in π–π stacking interactions between phenyl rings, while the longer sides participate in non-directional van der Waals contacts through *tert*-butyl groups, which fit into the voids formed by adjacent *tert*-butyl substituents from neighboring TBC6 molecules (Fig. 5 ▸).

The π–π stacking interactions are classified into two distinct types. In motif VI, the phenyl rings stack in a slightly shifted arrangement with hydroxyl groups positioned close to one another. In motif VII, the stacking is offset such that *tert*-butyl groups are brought into closer proximity. Motifs VI and VII alternate within a single plane [Fig. 5 ▸(*a*)]. On the longer sides of the macrocycle, the *tert*-butyl groups interdigitate in two characteristic ways: either with four interdigitating *tert*-butyl groups (motif VIII) or with two interacting groups (motif IX, Table S6).

The third guest solvent molecule (motif III) lies between these interdigitating *tert*-butyl motifs, occupying the interstitial space between columns. Intercolumnar solvent molecules are located between TBC6 molecules forming motif VI and are outside the TBC6 molecules in motif VII. Perpendicular to motifs VI (A⋯A) and VII (B⋯B), TBC6 molecules form motif VIII [Fig. 5 ▸(*b*)], a repetitive, alternating pattern C⋯D/D⋯C with one solvent molecule in between. In structure **7**, motif III is positioned differently: on one side near a *tert*-butyl group, and on the other stabilized by C—H⋯π interactions, bringing it closer to the TBC6 molecule.

**Figure 6:**
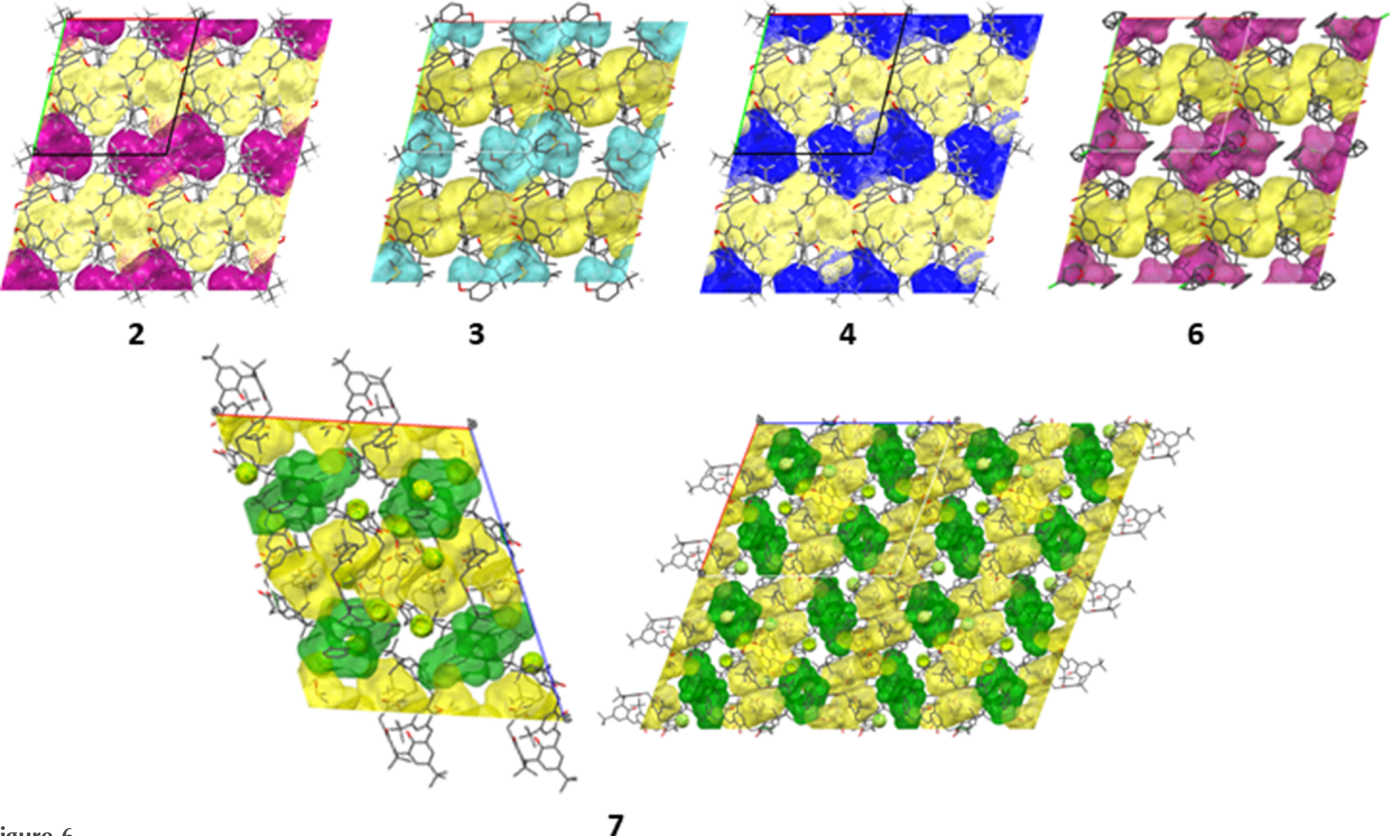
Crystal packing of structures **2**–**6** viewed along the *c* axis. Structure **7** is shown as the unit cell viewed along the *b* axis (left) and as a 2 × 2 × 2 supercell rotated by 69° counterclockwise (right). For clarity, H atoms are omitted; intracolumnar DMSO molecules are colored yellow. Other solvent species are colored as follows: **2**, DMF (pink); **3**, anisole (cyan); **4**, chlorobenzene and benzene (blue); **6**, 1,3-dichlorobenzene and THF (violet); **7**, 1,2-dichloro­benzene (green). Packing views along the *a*, *b* and *c* axes for all structures (**2**–**7**) are provided in Table S3.

Isostructurality was assessed using structure **2** as a reference, with heavy-atom overlays against structures **3**–**7**. RMSD values were: **2**–**3**: 0.09, **2**–**4**: 0.30, **2**–**5**: 0.59, **2**–**6**: 0.44 and **2**–**7**: 1.98. According to these results, structures **2**, **3** and **4** are unambiguously isostructural. The RMSD values obtained for structures **5** and **6** are somewhat higher but still within the range generally considered acceptable for isostructurality, and their packing motifs closely match that of structure **2** (Fig. 6 ▸). In contrast, structure **7** crystallizes in space group *P*2_1_/*n* with two TBC6 molecules, four DMSO molecules and two 1,2-di­chloro­benzene molecules in the asymmetric unit, and therefore cannot be regarded as isostructural with the others. Notably, structure **7** exhibits the highest solvent content, with guest molecules occupying 31.3% of the unit-cell volume (Table S9), yet it also shows the highest theoretical density among the analyzed structures (Table S1).

**Figure 7:**
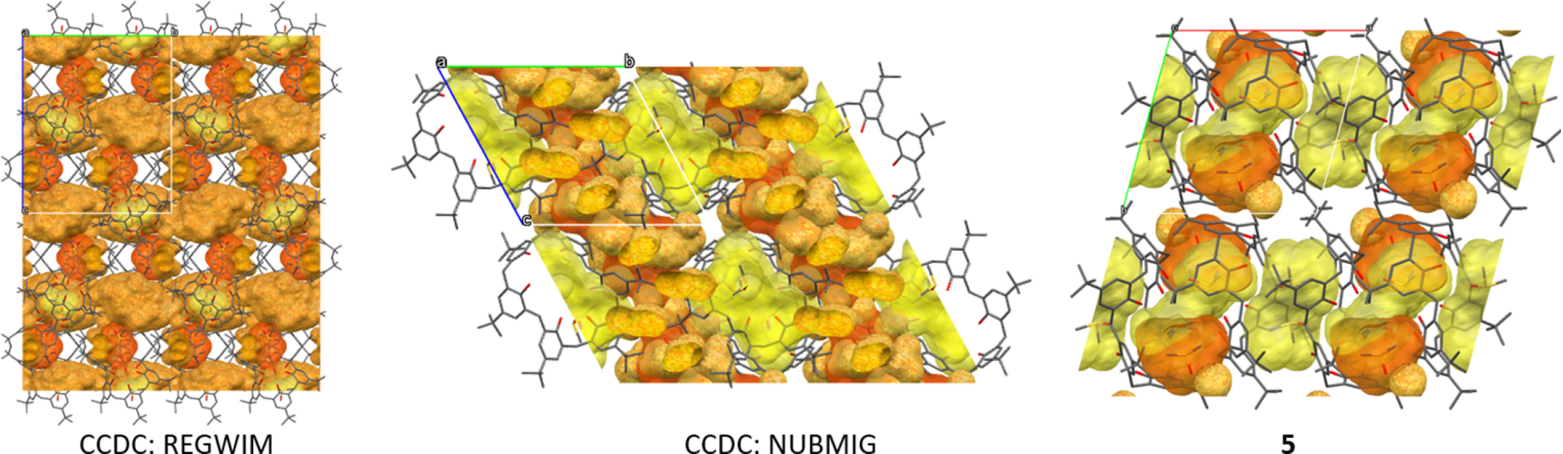
Crystal packing of the TBC6·DMSO solvates viewed along *a* for REGWIM and NUBMIG, and along *c* for **5**. For clarity, H atoms are omitted; intracolumnar DMSO molecules are colored yellow and intercolumnar DMSO molecules are colored orange. Packing views along *a*, *b* and *c* for all structures are provided in Table S3).

Structure **5** contains DMSO molecules in both intracolumnar and intercolumnar positions. It was also compared with two previously reported solvates of TBC6 with DMSO, both adopting the 1,2,3-alternate conformation: REGWIM (Martins *et al.*, 2017 ▸) and NUBMIG (Wolfgong *et al.*, 1996 ▸) (CCDC refcodes). In these previously reported structures, the TBC6:DMSO ratios are 1:2 and 2:5, respectively. Despite the differences in stoichiometry, structures **5** and NUBMIG are isostructural. In contrast, REGWIM forms similar TBC6–DMSO columns, but lacks intercolumnar DMSO molecules–in this case, only the columns are present, with no additional guest molecules occupying the space between them (Fig. 7 ▸, Table S4).

**Figure 8:**
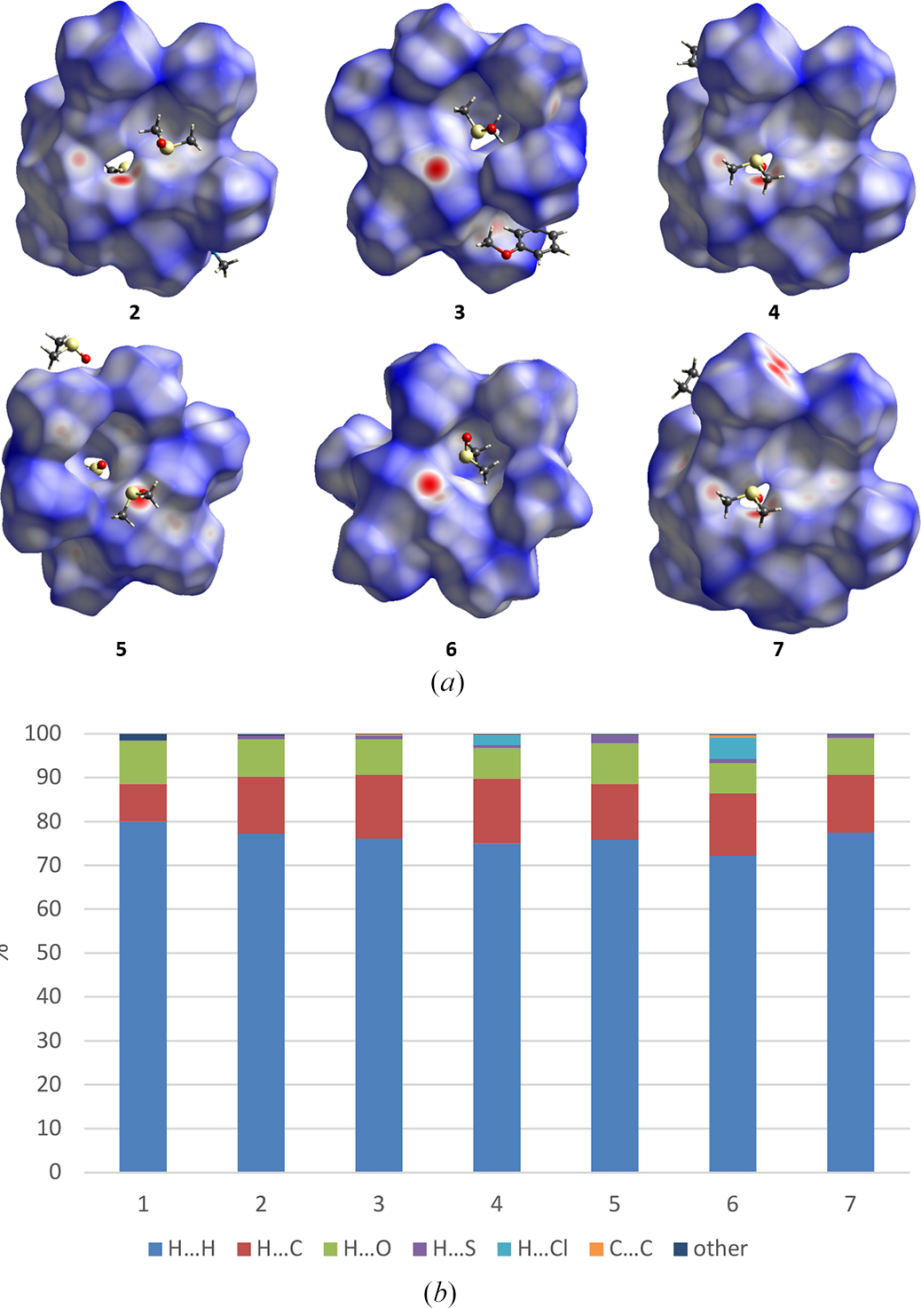
Interactions in the structures (*a*) visualized as Hirshfeld surfaces and (*b*) percentage contribution of particular contacts.

As expected, analysis of Hirshfeld surfaces [Figs. 8 ▸(*a*) and S4] showed that the strongest interactions occur for contacts between TBC6 and DMSO molecules via both, hydrogen bonds and C—H⋯π interactions, mostly through H⋯H, H⋯C and H⋯O contacts. [Fig. 8 ▸(*b*)] Interactions through C⋯S, O⋯S, N⋯H, Cl⋯O and O⋯O contacts have negligible contribution and were assigned as ‘other’. The average volume of Hirshfeld surface for structures that adopt 1,2,3-alternate conformation (**2**–**7**) is 1405 Å^3^ and the average surface area is equal to 1028 Å^2^.

### 3.2. Intermolecular interactions

The intermolecular interactions in the crystal structures of TBC6 solvates can be divided into TBC6–solvent motifs (I–III) and TBC6–TBC6 motifs (IV–IX). TBC6–solvent motifs are illustrated in Fig. 3 ▸, where motifs I and I′ involve hydrogen bonding, while motifs II and II′ represent C—H⋯π interactions within the TBC6 cavity. These interactions are confined within columns. Motif III corresponds to the interaction between TBC6 and an intercolumnar guest molecule.

**Figure 9:**
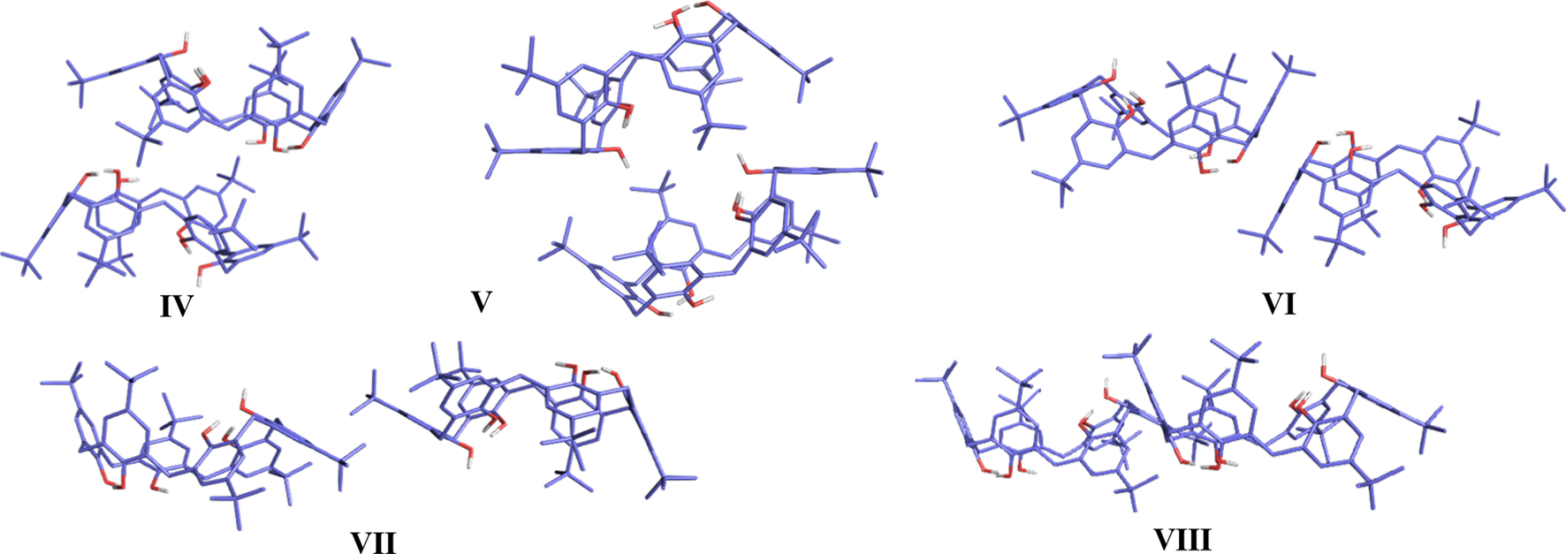
Selected TBC6–TBC6 structural motifs from the TBC6 solvates with 1,2,3-alternate conformation.

TBC6–TBC6 interactions, presented in Fig. 9 ▸ and Table S6, include intra-column interactions (motifs IV and V) and inter-column interactions (motifs VI–IX). Due to the distinct conformation of structure **1**, its interactions are discussed separately (Fig. S3).

The calculated interaction energies (Tables 2 ▸ and S5) show that motif II′ (C—H⋯π interaction) is the strongest TBC6–guest interaction for structures **2**–**7**, with energies consistently below −80 kJ mol^−1^ (Table 2 ▸). This interaction is stronger than both hydrogen-bonding motifs (I and I′). Among the hydrogen-bonded motifs, motif I′, which involves shorter hydrogen-bond distances (Tables S11–S16), is consistently stronger than motif I. Notably, motifs I′ and II′ are formed between symmetry-equivalent molecules.

Motif III, representing the intercolumnar guest, shows moderate stabilization, with the most favorable energy observed for chloro­benzene in structure **4** (−38.9 kJ mol^−1^). Other solvents yield similar interaction strengths, typically ranging from −31.3 to −38.9 kJ mol^−1^. Although these interactions are weaker compared to other motifs, they are consistent across all solvents studied.

Among the TBC6–TBC6 interactions, motifs IV and V (intra-columnar) are generally the most stabilizing. Motif IV is the strongest interaction in structure **5** (−95.5 kJ mol^−1^), while motif V is the dominant interaction in structures **2**, **3**, **6** and **7**, with values as low as −102.0 kJ mol^−1^ (structure **2**). These strong stabilizing interactions explain why columnar packing is a common feature across all these structures.

In contrast, inter-column interactions (motifs VI–IX) are significantly weaker, typically about half as strong as intra-column interactions, which may account for the presence of the third solvent molecule between columns. Motifs VII–IX show relatively modest contributions. For example, motif VII ranges from −23.5 to −33.9 kJ mol^−1^, and motif IX from −18.9 to −26.4 kJ mol^−1^.

### 3.3. Influence of solvent properties on the crystal forms of TBC6

Previous studies demonstrated that TBC6 can purify solvent mixtures by selectively removing chloro­benzene, with solvent size playing a crucial role in molecular recognition (Zaorska *et al.*, 2023 ▸). In this work, we present analogous examples using solvent mixtures containing DMSO. Our results indicate that DMSO competes effectively with other solvents, including chloro­benzene; though its selectivity is incomplete. To elucidate these trends, we analyzed solvent properties such as density, relative polarity, dipole moment, dielectric constant, molecular weight, topological polar surface area, and standard enthalpy of formation at 298 K, as summarized in Table S8. No clear trends were observed for the other properties considered beyond density and enthalpy.

For crystallizations involving mixtures of up to four solvents (**2**, **3**, **5** and **6**), the intercolumnar guest molecule consistently exhibits a higher density than DMSO. For example, in **5** (DMSO/ethanol), ethanol—a protic solvent that typically does not form stable complexes with TBC6—was excluded from the intercolumnar site due to its lower density (0.81 g cm^−3^ versus 0.99 g cm^−3^ for DMSO). In **3** (DMSO/anisole/*n*-heptane), the similar densities of DMSO (0.94 g cm^−3^) and anisole (0.99 g cm^−3^) led to their co-occupancy in the intercolumnar space (total occupancy = 1).

A second trend emerged for the standard enthalpy of formation Δ_f_*H*^o^_298 K_(liquid): intercolumnar guests generally had higher Δ_f_*H*^o^_298 K_(liquid) values than DMSO (−239 kJ mol^−1^). For instance, in **5**, DMSO (higher Δ_f_*H*^o^_298 K_(liquid) than ethanol) occupied the intercolumnar site. These trends were partially observed in **4** and **6** (crystallized from 12- and 16-solvent mixtures, respectively), where intercolumnar sites exhibited substitutional disorder. In **4**, chloro­benzene [higher density, Δ_f_*H*^o^_298 K_(liquid) = 11.5 kJ mol^−1^] and benzene coexisted, while **6** contained 1,3-di­chloro­benzene and THF. Notably, THF deviated from the trend due to its larger dipole moment and dielectric constant.

Chloro­benzene’s selectivity in prior work correlated with its high Δ_f_*H*^o^_298 K_(liquid), suggesting this parameter influences solvate formation. While DMSO’s low Δ_f_*H*^o^_298 K_(liquid) does not inherently favor its incorporation, its competitiveness is evident in **4** (chloro­benzene occupancy = 0.54) and its exclusion in **6**. We propose that solvents with higher Δ_f_*H*^o^_298 K_(liquid) may stabilize the crystal structure by reducing the system’s overall energy, though further investigation is needed.

## 4. Conclusions

We report seven new TBC6 solvate structures, revealing two conformational states: the winged cone (**1**) and the 1,2,3-alternate form (**2**–**7**). In the latter, each TBC6 unit hosts three guest molecules—two intracolumnar DMSO molecules and one intercolumnar guest—yielding a consistent columnar packing motif stabilized by hydrogen bonds (motifs I/I′) and C—H⋯π interactions (motifs II/II′). The strongest TBC6–guest interaction involves a DMSO molecule engaged in C—H⋯π contacts (<−80 kJ mol^−1^), with concomitant hydrogen bonding explaining its retention up to ∼200°C (versus 120–150°C for other guests).

Hirshfeld surface analysis confirmed that TBC6–TBC6 interactions dominate crystal stability, particularly intra­columnar motifs IV/V, which are twice as stabilizing as intercolumnar motifs VI–IX. This energy disparity rationalizes the inclusion of intercolumnar solvent guests. These results further demonstrate that conformations calculated to be higher in intrinsic energy, such as the winged-cone and 1,2,3-alternate forms, can nevertheless be stabilized in the solid state by favorable host–guest interactions and crystal packing effects. Beyond solvent size, density and Δ_f_*H*^o^_298 K_(liquid) emerged as potential predictors of guest selectivity, though kinetic factors (*e.g.* winged-cone formation) also play a role.

## Supplementary Material

Crystal structure: contains datablock(s) 1, 2, 3, 4, 5, 6, 7. DOI: 10.1107/S2052520625008625/um5068sup1.cif

Structure factors: contains datablock(s) 1. DOI: 10.1107/S2052520625008625/um50681sup3.hkl

Structure factors: contains datablock(s) 2. DOI: 10.1107/S2052520625008625/um50682sup4.hkl

Structure factors: contains datablock(s) 3. DOI: 10.1107/S2052520625008625/um50683sup5.hkl

Structure factors: contains datablock(s) 4. DOI: 10.1107/S2052520625008625/um50684sup6.hkl

Structure factors: contains datablock(s) 5. DOI: 10.1107/S2052520625008625/um50685sup7.hkl

Structure factors: contains datablock(s) 6. DOI: 10.1107/S2052520625008625/um50686sup8.hkl

Structure factors: contains datablock(s) 7. DOI: 10.1107/S2052520625008625/um50687sup9.hkl

Tables S1-S16, Figs S1-S4. DOI: 10.1107/S2052520625008625/um5068sup2.pdf

CCDC references: 2446418, 2446419, 2446420, 2446421, 2446422, 2446423, 2446424

## Figures and Tables

**Table:** Experiments were carried out at 100 K with Cu *K*α radiation using a SuperNova, Dual, Cu at home/near, HyPix. Full experimental details of data collection, data reduction, and data refinement of **1**–**7** are available in Table S1 and in the CIF.

	**1**	**2**	**3**	**4**
Formula	2TBC6·2DMSO·8DMSO	TBC6·2DMSO·DMF	TBC6·2DMSO·0.66DMSO·0.34anisole	TBC6·2DMSO·0.63chlorobenzene·0.37benzene
Chemical formula	2C_2_H_6_OS·2C_66_H_84_O_6_·8C_2_H_6_SO	2C_2_H_6_OS·C_66_H_84_O_6_·C_3_H_7_NO	2.662C_2_H_6_OS·C_66_H_84_O_6_·0.338C_7_H_8_O	2C_2_H_6_OS·0.634C_6_H_5_Cl·C_66_H_84_O_6_·0.366C_6_H_6_
*M*_r_	2102.91	1202.68	1217.79	1229.56
Crystal system, space group	Triclinic, *P*1	Triclinic, *P*1	Triclinic, *P*1	Triclinic, *P*1
*a*, *b*, *c* (Å)	13.3001 (6), 14.4711 (7), 22.2286 (14)	15.7313 (3), 15.9323 (3), 17.2805 (3)	15.6856 (2), 16.1397 (2), 17.2786 (2)	15.8827 (3), 16.0420 (3), 17.2929 (3)
α, β, γ (°)	76.056 (5), 74.698 (5), 74.565 (4)	112.7464 (18), 116.3321 (18), 91.3671 (15)	113.002 (1), 116.277 (1), 91.356 (1)	112.768 (2), 116.199 (2), 91.653 (1)
*V* (Å^3^)	3910.1 (4)	3478.37 (13)	3506.41 (8)	3537.57 (13)
*Z*	1	2	2	2

Refinement				
*R*[*F*^2^ > 2σ(*F*^2^)], *wR*(*F*^2^), *S*	0.088, 0.243, 1.03	0.065, 0.199, 1.08	0.057, 0.163, 1.07	0.043, 0.117, 1.06
No. of reflections	15380	13696	14757	14895
No. of parameters	1284	827	886	864
No. of restraints	1051	84	33	38
Δρ_max_, Δρ_min_ (e Å^−3^)	0.41, −0.38	1.54, −1.63	0.86, −0.90	0.75, −0.53
CCDC number	2446419	2446418	2446422	2446424

**Table:** 

	**5**	**6**	**7**
Formula	TBC6·3DMSO	TBC6·2DMSO·0.41(1,3-dichloro­benzene)·0.59THF	2TBC6·4DMSO·2(1,2-dichloro­benzene)
Chemical formula	3C_2_H_6_OS·C_66_H_84_O_6_	2C_2_H_6_OS·C_66_H_81.05_O_6_·C_4.835_H_6.33_Cl_0.835_O_0.582_	C_6_H_4_Cl_2_·2C_2_H_6_OS·C_66_H_84_O_6_
*M*_r_	1207.71	1229.94	1276.57
Crystal system, space group	Triclinic, *P* 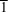	Triclinic, *P* 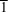	Monoclinic, *P*2_1_/*n*
*a*, *b*, *c* (Å)	15.9943 (4), 16.1631 (4), 16.9876 (4)	15.7755 (3), 16.1225 (3), 17.2814 (2)	27.75588 (14), 16.68904 (7), 33.30961 (18)
α, β, γ (°)	117.382 (3), 113.315 (3), 91.961 (2)	113.063 (1), 116.216 (2), 91.229 (1)	90, 111.0531 (6), 90
*V* (Å^3^)	3452.93 (18)	3525.90 (12)	14399.68 (13)
*Z*	2	2	8

*R*[*F*^2^ > 2σ(*F*^2^)], *wR*(*F*^2^), *S*	0.052, 0.146, 1.05	0.109, 0.316, 1.04	0.037, 0.101, 1.04
No. of reflections	13550	13866	30310
No. of parameters	787	776	1736
No. of restraints	0	38	1
Δρ_max_, Δρ_min_ (e Å^−3^)	0.65, −0.57	4.85, −1.98†	0.56, −0.53
CCDC number	2446420	2446421	2446423

† High residual density peaks suggest the presence of an additional solvent molecule; however, it could not be modeled as an atomic site and lies too close to the 1,3-di­chloro­benzene and THF molecules to allow the use of a solvent-mask procedure.

**Table 2:** Interaction energies (in kJ mol^−1^) for selected TBC6–solvent (I–III) and TBC6–TBC6 (IV–IX) structural motifs in crystal structures **1**–**7** Due to differences in molecular conformation and crystal packing, the TBC6–solvent and TBC6–TBC6 motifs differ between structure **1** and structures **2**–**7**, as shown in Figs. S3 and 9 ▸, respectively. Motifs I′, II and II′ are absent in structure **1**. Some motifs in structure **7** occur twice because multiple independent molecules are present in the asymmetric unit. Pairwise interaction energies were computed with the counterpoise correction in *Gaussian* at the B3LYP/6-31G(d,p) level with added 6d/10f polarization functions.

		Motifs									
	Guest	I	I′	II	II′	III	IV	V	VI	VII	VIII
**1**	DMSO	−120.2	n/a	n/a	n/a	n/a	−90.9	−45.9	−20.7	−19.1	−33.3
**2**	DMSO:DMF (2:1)	−67.3	−80.6	−49.7	−80.1	−38.8	−71.9	−102.0	−45.2	−24.6	−27.4
**3**	DMSO:anisole (2:1)	−66.9	−79.6	−50.2	−80.7	−37.4	−88.4	−95.9	−44.4	−23.5	−22.3
**4**	DMSO:chloro­benzene (2:1)	−66.8	−78.5	−51.3	−83.7	−38.9	−78.3	−93.8	−38.3	−24.2	−26.2
**5**	DMSO	−67.6	−72.7	−54.3	−86.4	−37.2	−95.5	−82.7	−52.7	−33.9	−25.4
**6**	DMSO:THF (2:1)	−59.6	−77.1	−53.0	−84.8	−31.3	−91.5	−97.7	−43.4	−24.4	−25.9
**7**	DMSO:1,2-di­chloro­benzene (2:1)	−68.3	−79.0	−52.2	−79.6	−36.9	−87.1	−92.3	−52.6	−33.6	−26.8
		−70.9	−72.9	−61.8	−80.6	−36.3		−93.3	−66.6		
