# Supplementary material for: DMSO solvates of tert-butyl­calix[6]arene and related multisolvent structures
Source: Acta Crystallogr B Struct Sci Cryst Eng Mater. 2025 Nov 5;81(Pt 6):517–27. doi: 10.1107/S2052520625008625 (PMC12786388; doi:10.1107/S2052520625008625)
Supplement: Supplementary file 9 [file b-81-00517-sup2.pdf]

STRUCTURAL SCIENCE
CRYSTAL ENGINEERING
MATERIALS

Volume 81 (2025)

Supporting information for article:

DMSO solvates of *tert*-butylcalix[6]arene and related multisolvent structures

Monika Wanat, Ewelina Zaorska and Maura Malinska

Table S1 Experimental details of data collection, data reduction, and data refinement of **1-7**. Experiments were carried out at 100 K with Cu Kα radiation using a SuperNova, Dual, Cu at home/near, HyPix.

	**1**	**2**	**3**	**4**
Crystal data				
Chemical formula	$2(C_2H_6OS)·2(C_{66}H_{84}O_6)$·8[C2H6SO]	$2(C_2H_6OS)·C_{66}H_{84}O_6·C_3H_7NO$	$2.662(C_2H_6OS)·C_{66}H_{84}O_6·0.338(C_7H_8O)$	$2(C_2H_6OS)·0.634(C_6H_5Cl)·C_{66}H_{84}O_6·0.366(C_6H_6)$
M_r	2102.91	1202.68	1217.79	1229.56
Crystal system, space group	Triclinic, *P*1	Triclinic, $P\bar{1}$	Triclinic, $P\bar{1}$	Triclinic, $P\bar{1}$
a, *b*, *c* (Å)	13.3001 (6), 14.4711 (7), 22.2286 (14)	15.7313 (3), 15.9323 (3), 17.2805 (3)	15.6856 (2), 16.1397 (2), 17.2786 (2)	15.8827 (3), 16.0420 (3), 17.2929 (3)
α, β, γ (°)	76.056 (5), 74.698 (5), 74.565 (4)	112.7464 (18), 116.3321 (18), 91.3671 (15)	113.002 (1), 116.277 (1), 91.356 (1)	112.768 (2), 116.199 (2), 91.653 (1)
V (Å^3)	3910.1 (4)	3478.37 (13)	3506.41 (8)	3537.57 (13)
Z	1	2	2	2
m (mm^{-1})	0.68	1.12	1.29	1.31
Crystal size (mm)	0.26 × 0.17 × 0.03	0.22 × 0.21 × 0.15	0.24 × 0.17 × 0.03	0.18 × 0.14 × 0.04
Data collection				
Absorption correction	Gaussian *CrysAlis PRO* 1.171.42.36a (Rigaku Oxford Diffraction, 2021) Numerical absorption correction based on gaussian integration over a multifaceted crystal model Empirical absorption correction using spherical harmonics, implemented in SCALE3 ABSPACK scaling algorithm.	Multi-scan *CrysAlis PRO* 1.171.42.36a (Rigaku Oxford Diffraction, 2021) Empirical absorption correction using spherical harmonics, implemented in SCALE3 ABSPACK scaling algorithm.	Multi-scan	Multi-scan *CrysAlis PRO* 1.171.42.70a (Rigaku Oxford Diffraction, 2022) Empirical absorption correction using spherical harmonics, implemented in SCALE3 ABSPACK scaling algorithm.

T_{min}, T_{max}	0.693, 1.000	0.663, 1.000	0.934, 1.000	0.893, 1.000
No. of measured, independent and observed [$I > 2\sigma(I)$] reflections	36778, 15380, 11749	66517, 13696, 11955	72404, 14757, 12578	72678, 14895, 12786
R_{int}	0.082	0.038	0.038	0.032
θ_{max} (°)	51.3	72.1	77.9	77.8
$(\sin \theta/\lambda)_{max}$ (Å^{-1})	0.506	0.617	0.634	0.634
Refinement				
$R[F^2 > 2\sigma(F^2)]$, $wR(F^2)$, S	0.088, 0.243, 1.02	0.065, 0.199, 1.08	0.057, 0.163, 1.07	0.043, 0.117, 1.06
No. of reflections	15380	13696	14757	14895
No. of parameters	1300	827	886	864
No. of restraints	1051	84	33	38
H-atom treatment	H-atom parameters constrained	H-atom parameters constrained	H-atom parameters constrained	H-atom parameters constrained
	$w = 1/[\sigma^2(F_o^2) + (0.1759P)^2]$ where $P = (F_o^2 + 2F_c^2)/3$	$w = 1/[\sigma^2(F_o^2) + (0.1137P)^2 + 3.3243P]$ where $P = (F_o^2 + 2F_c^2)/3$	$w = 1/[\sigma^2(F_o^2) + (0.0842P)^2 + 2.105P]$ where $P = (F_o^2 + 2F_c^2)/3$	$w = 1/[\sigma^2(F_o^2) + (0.0531P)^2 + 1.6269P]$ where $P = (F_o^2 + 2F_c^2)/3$
$\Delta\rho_{max}$, $\Delta\rho_{min}$ (e Å^{-3})	0.41, -0.38	1.54, -1.63	0.86, -0.90	0.75, -0.53
Absolute structure	Flack x determined using 3901 quotients [(I+)-(I-)]/[(I+)+(I-)] (Parsons, Flack and Wagner, Acta Cryst. B69 (2013) 249-259).	–	–	–
Absolute structure parameter	0.48 (4)	–	–	–

	**5**	**6**	**7**
Crystal data			
Chemical formula	$3(C_2H_6OS)\cdot C_{66}H_{84}O_6$	$2(C_2H_6OS)\cdot C_{66}H_{81.05}O_6\cdot C_{4.835}H_{6.33}Cl_{0.835}O_{0.582}$	$C_6H_4Cl_2\cdot 2(C_2H_6OS)\cdot C_{66}H_{84}O_6$
M_r	1207.71	1229.94	1276.57
Crystal system, space group	Triclinic, $P\bar{1}$	Triclinic, $P\bar{1}$	Monoclinic, $P2_1/n$
a, b, c (Å)	15.9943 (4), 16.1631 (4), 16.9876 (4)	15.7755 (3), 16.1225 (3), 17.2814 (2)	27.75588 (14), 16.68904 (7), 33.30961

			(18)
α, β, γ (°)	117.382 (3), 113.315 (3), 91.961 (2)	113.063 (1), 116.216 (2), 91.229 (1)	90, 111.0531 (6), 90
V (Å^3)	3452.93 (18)	3525.90 (12)	14399.68 (13)
Z	2	2	8
m (mm^{-1})	1.40	1.39	1.76
Crystal size (mm)	0.20 × 0.05 × 0.05	0.37 × 0.11 × 0.04	0.22 × 0.18 × 0.10
Data collection			
Absorption correction	Multi-scan *CrysAlis PRO* 1.171.42.70a (Rigaku Oxford Diffraction, 2022) Empirical absorption correction using spherical harmonics, implemented in SCALE3 ABSPACK scaling algorithm.	Multi-scan *CrysAlis PRO* 1.171.42.70a (Rigaku Oxford Diffraction, 2022) Empirical absorption correction using spherical harmonics, implemented in SCALE3 ABSPACK scaling algorithm.	Multi-scan *CrysAlis PRO* 1.171.42.70a (Rigaku Oxford Diffraction, 2022) Empirical absorption correction using spherical harmonics, implemented in SCALE3 ABSPACK scaling algorithm.
T_{min}, T_{max}	0.807, 1.000	0.536, 1.000	0.741, 1.000
No. of measured, independent and observed [$I > 2\sigma(I)$] reflections	62371, 13550, 9828	64928, 13866, 11028	150374, 30310, 26207
R_{int}	0.062	0.072	0.032
θ_{max} (°)	73.2	73.0	77.7
$(\sin \theta/\lambda)_{max}$ (Å^{-1})	0.621	0.620	0.634
Refinement			
$R[F^2 > 2\sigma(F^2)]$, $wR(F^2)$, S	0.052, 0.146, 1.05	0.109, 0.316, 1.04	0.037, 0.101, 1.04
No. of reflections	13550	13866	30310
No. of parameters	787	776	1736
No. of restraints	0	38	1
H-atom treatment	H-atom parameters constrained	H atoms treated by a mixture of independent and constrained refinement	H atoms treated by a mixture of independent and constrained refinement
	$w = 1/[\sigma^2(F_o^2) + (0.0701P)^2 + 1.2302P]$ where $P = (F_o^2 + 2F_c^2)/3$	$w = 1/[\sigma^2(F_o^2) + (0.1799P)^2 + 10.0354P]$ where $P = (F_o^2 + 2F_c^2)/3$	$w = 1/[\sigma^2(F_o^2) + (0.0477P)^2 + 6.9048P]$ where $P = (F_o^2 + 2F_c^2)/3$
$\Delta\rho_{max}$, $\Delta\rho_{min}$ (e Å^{-3})	0.65, -0.57	4.85, -1.98	0.56, -0.52

Computer programs: *CrysAlis PRO* 1.171.42.36a (Rigaku OD, 2021), *CrysAlis PRO* 1.171.42.70a (Rigaku OD, 2022), *SHELXD* (Sheldrick, 2008), *SHELXT* 2018/2 (Sheldrick, 2018), olex2.solve 1.5

(Bourhis *et al.*, 2015), *SHELXT* 2014/5 (Sheldrick, 2014), *SHELXL* 2019/2 (Sheldrick, 2015), Olex2 1.5 (Dolomanov *et al.*, 2009).

Table S2 Comparison of geometry of TBC6 molecular structures **2-7**.

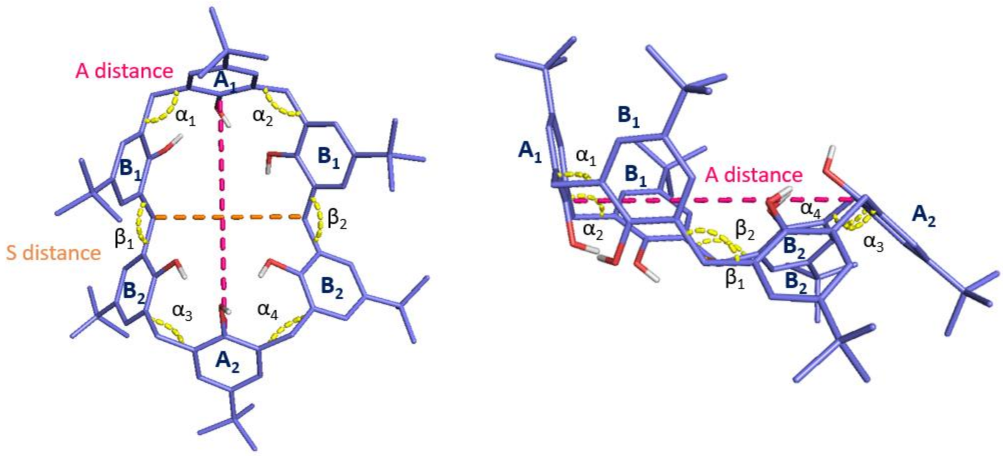

TBC6	intercolumnar guests	S distance [Å]	A distance [Å]	α1 [º]	α2 [º]	α3 [º]	α4 [º]	β1 [º]	β2 [º]
**2**	DMF	6.065(3)	9.152(4)	112.0(4)	113.5(2)	113.5(2)	116.4(2)	117.1(2)	116.4 (2)
**3**	0.66•DMSO 0.34• anisole	5.993(3)	9.185(3)	112.89(19)	112.60(15)	116.13(16)	112.7(2)	116.61(16)	117.6(2)
**4**	0.63• chlorobenzene 0.37•benzene	5.932(2)	9.172(3)	111.9(12)	113.3(2)	112.6(2)	115.8(1)	117.8(2)	115.8(1)
**5**	DMSO	6.177(3)	8.983(3)	112.9(2)	111.9(2)	111.7(2)	112.4(3)	117.6(2)	116.8(3)
**6**	0.41•1,4-di chlorobenzene 0.59•THF	5.933(6)	9.185(8)	112.0(3)	113.2(4)	112.7(4)	116.1(4)	116.8(4)	115.9(4)
**7**	1,2-dichlorobenzene	6.2299(9) 5.8268(19)	9.0672(16) 9.2792(16)	113.43(10) 113.08(11)	112.45(11) 110.80(9)	113.28(12) 114.44(10)	114.57(10) 114.37(12)	114.57(10) 116.99(11)	118.59(10) 114.17(13)

Table S3 Columns in structures **1-7**, REGWIM, NUBMIG, REGWEI – views from the top and from the side. For the clarity, views of **6** and REGWIM are not along any axis.

	Columns: view from the top	Columns: view from the side
**1**	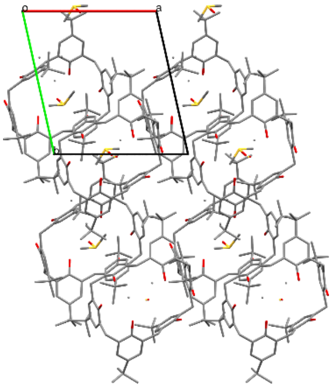	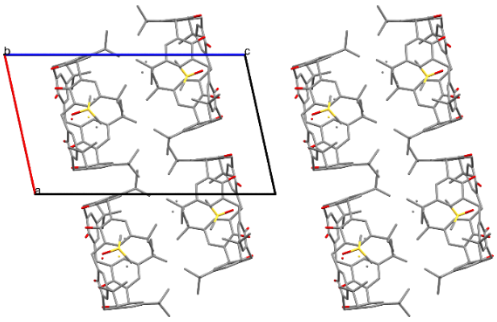
**2**	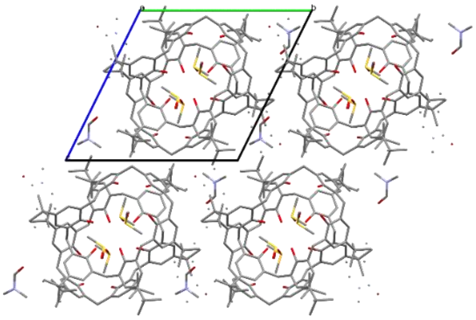	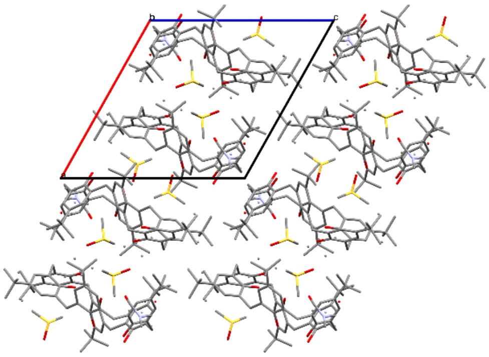
**3**	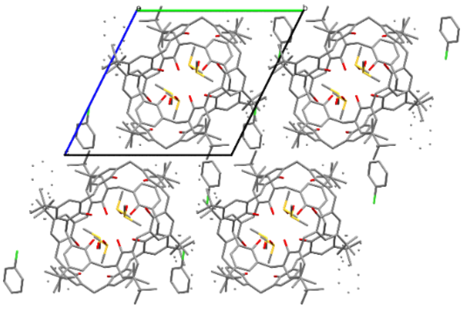	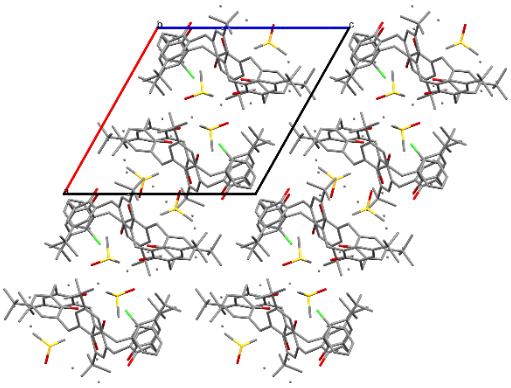
**4**	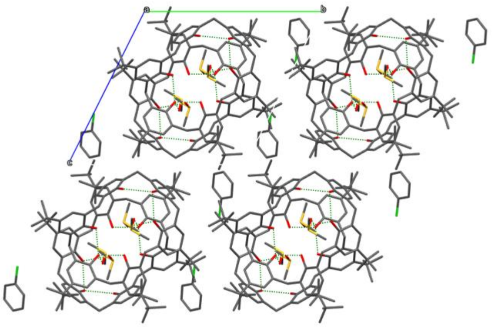	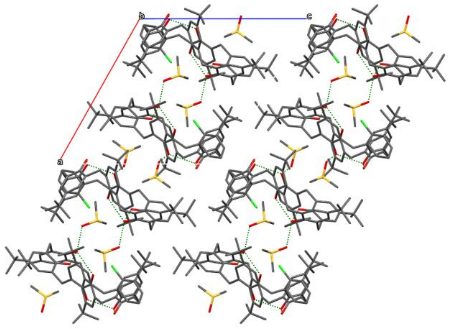

5

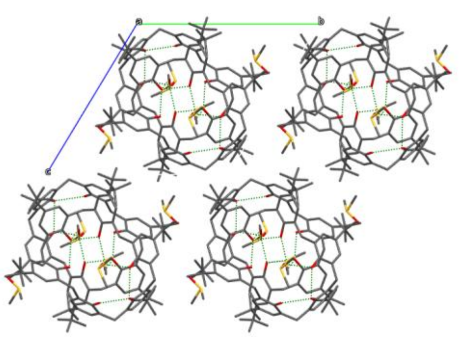

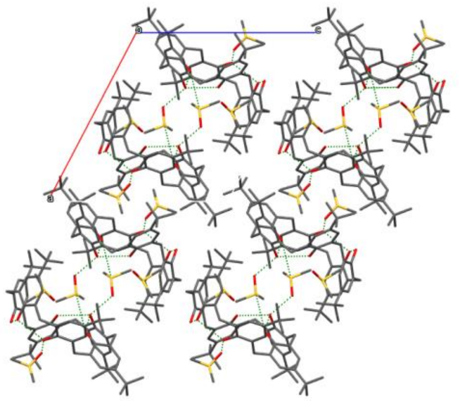

6

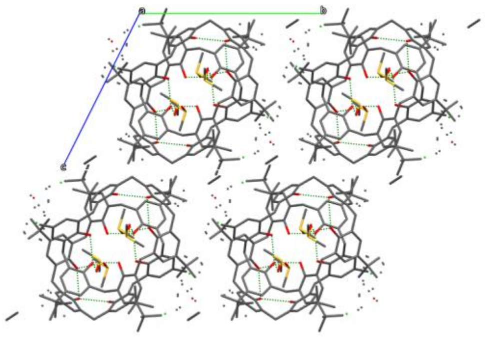

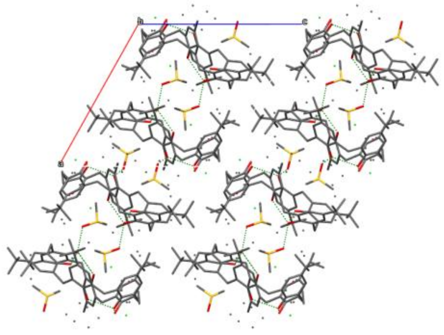

7

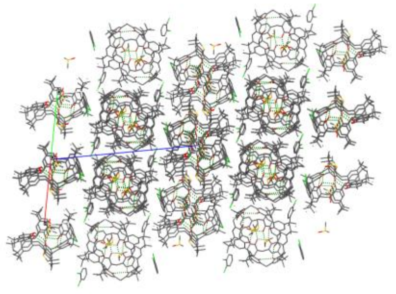

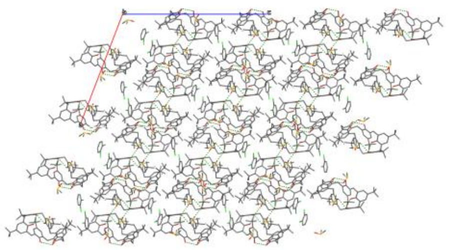

REGWIM

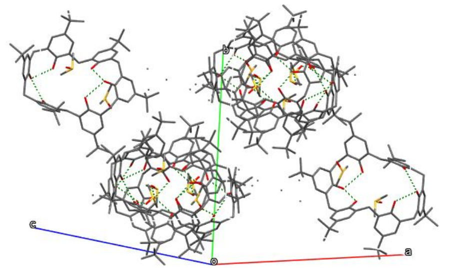

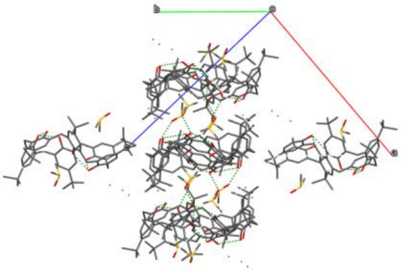

NUBMIG

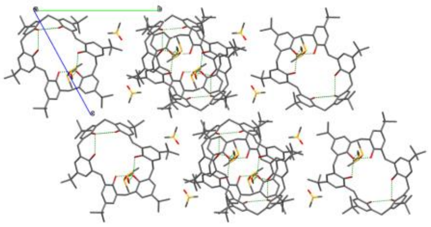

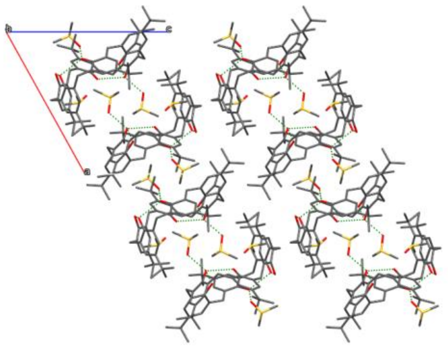

REGWEI

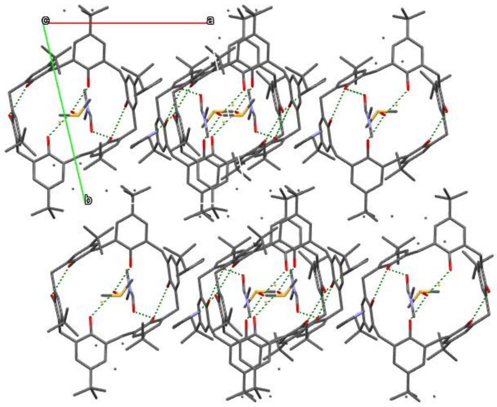

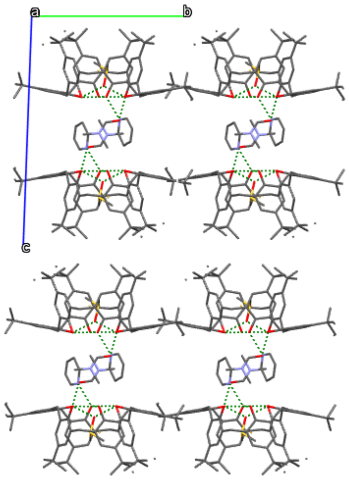

Table S4 Crystal packing of the analysed calix[6]arene solvates along X, Y, and Z axis. For clarity, hydrogen atoms are omitted and intracolumnar DMSO molecules are assigned with yellow colour. The other solvents are colored as follow: **2**) pink – DMF, **3**) cyan – DMSO and anisole **4**) blue – chlorobenzene and benzene, **5**) orange – intercolumnar DMSO , **6**) green – 1,2-dichlorobenzene, **7**) violet – 1,3-dichlorobenzene and THF, **REGWEI**) for clarity both intermolecular solvents (DMF and pyridine) are assigned with pink, **REGWIM)** two intramolecular DMSO occur, for clarity one of them is in orange. **NUBMIG**

	a	*b*	*c*
**1**	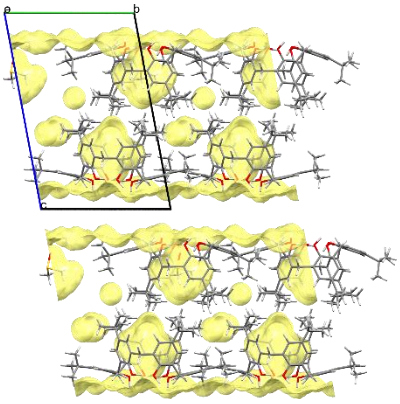	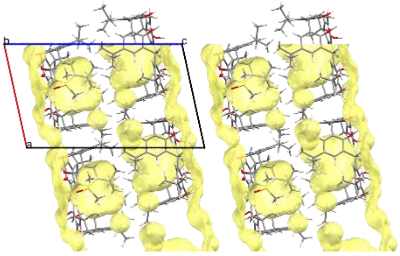	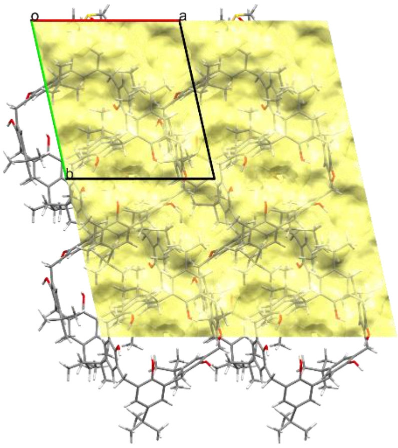
**CCDC: REGWEI**	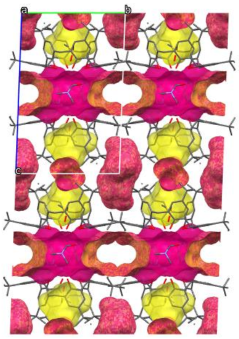	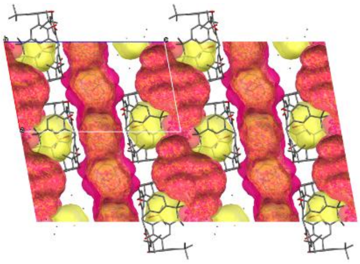	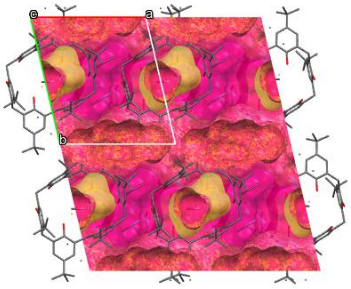
**2**	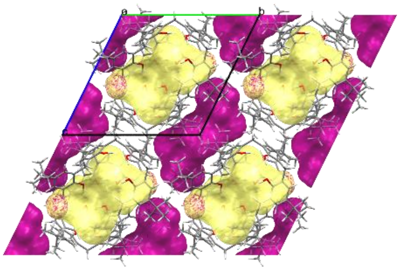	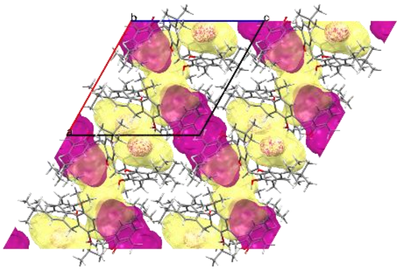	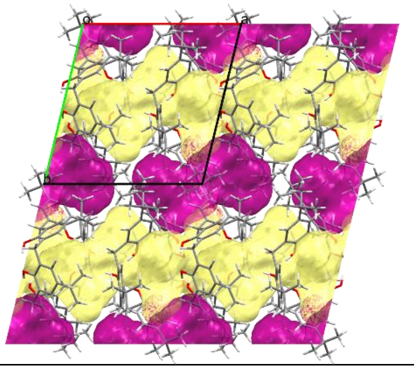

**3**	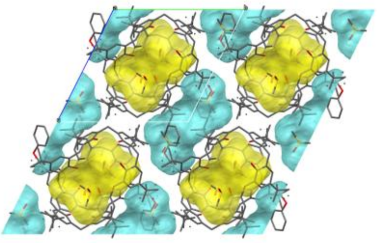	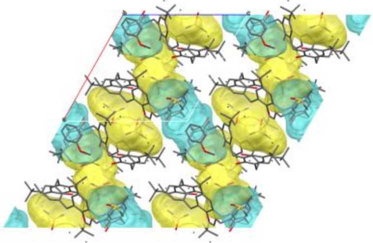	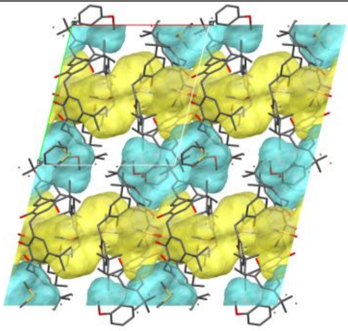
**4**	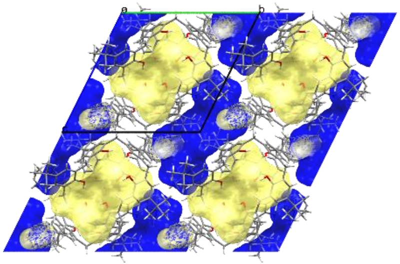	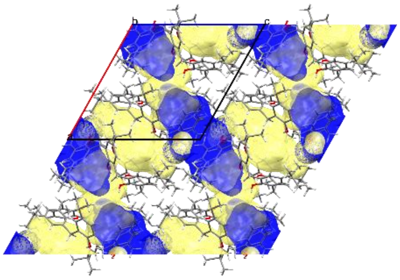	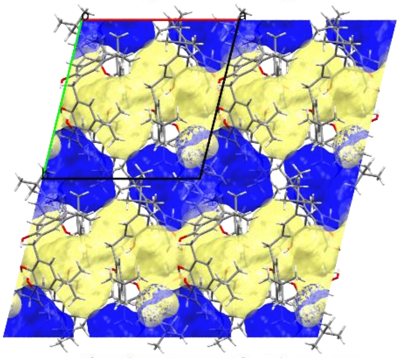
**5**	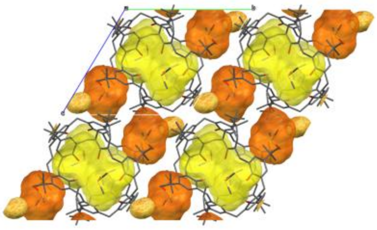	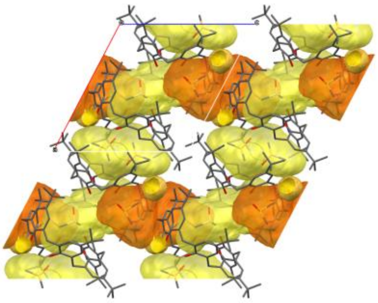	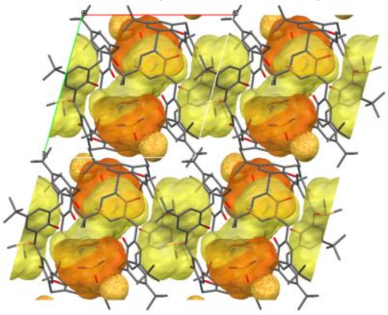
**CCDC: REGWIM**	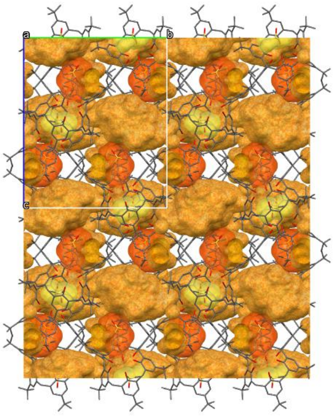	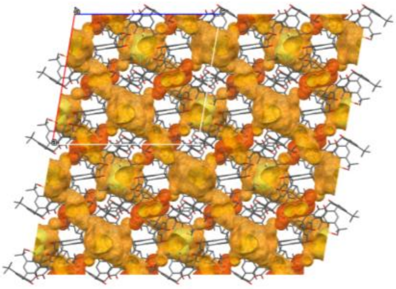	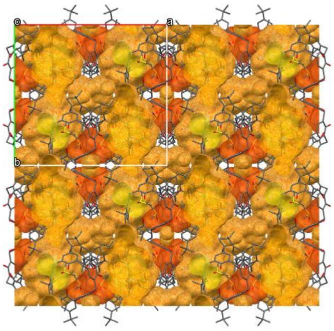

**CCDC: NUBMIG**	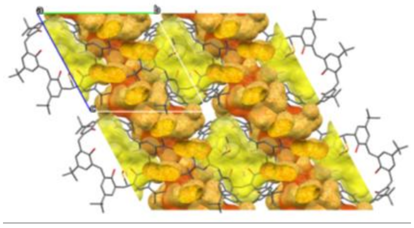	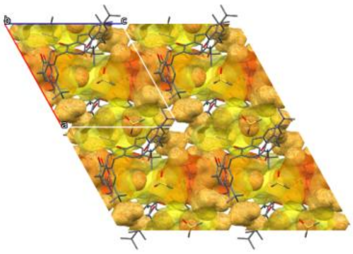	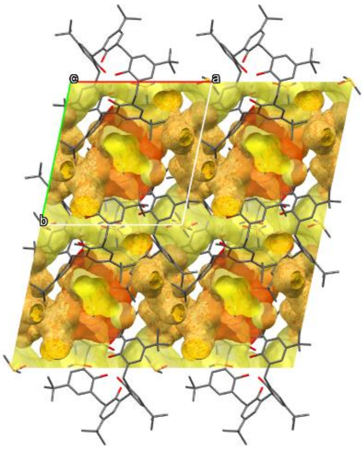
**6**	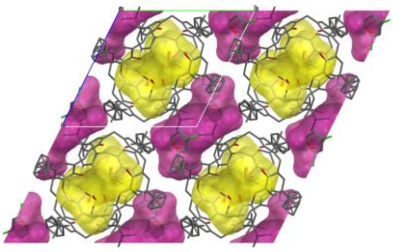	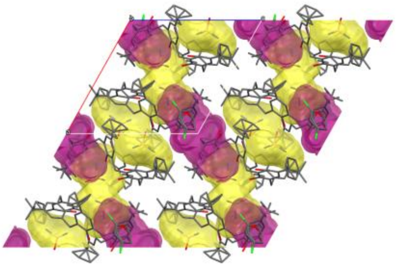	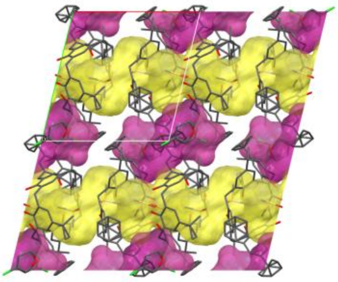
**7**	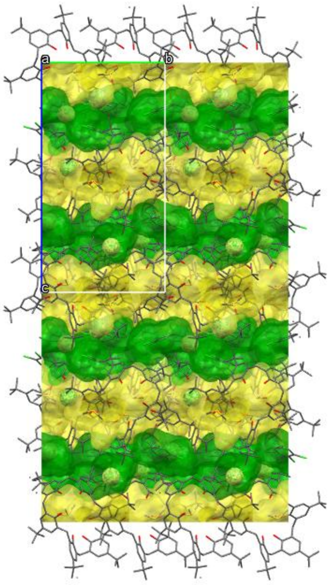	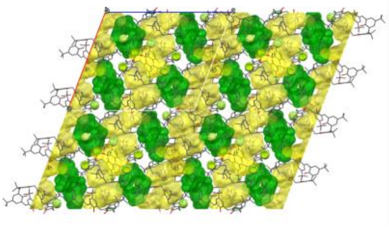	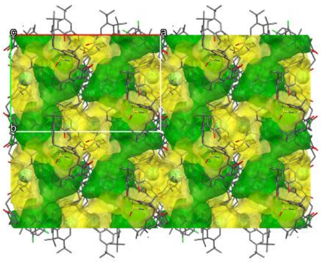

Table S5 Interaction energies presented in kJ·mol^{-1} for selected TBC6–guest (I–III) and TBC6–TBC6 (II–VIII) structural motifs in the analysed crystals **1-7**. Due to conformation, the TBC6-TBC6 structural motifs are different for **1** and **2-7** – they are presented at Fig. S3 and Fig. 6, respectively. Calculations performed in Gaussian program using counterpoise method with the theory level B3LYP/6-31G(d,p)_6d_10f.

		Motifs										
	Guest	I	I’	II	II’	III	IV	V	VI	VII	VIII	IX
**1**	DMSO	-120.2	n/a	n/a	n/a	n/a	-90.9	-45.9	-20.7	-19.1	-33.3	
**2**	DMSO: DMF (2:1)	-67.3	-80.6	-49.7	-80.1	-38.8	-71.9	-102.0	-45.2	-24.6	-27.4	-26.4
**3**	DMSO: anisole (2:1)	-66.9	-79.6	-50.2	-80.7	-37.4	-88.4	-95.9	-44.4	-23.5	-22.3	-22.4
**4**	DMSO: chlorobenzene (2:1)	-66.8	-78.5	-51.3	-83.7	-38.9	-78.3	-93.8	-38.3	-24.2	-26.2	-24.3
**5**	DMSO	-67.6	-72.7	-54.3	-86.4	-37.2	-95.5	-82.7	-52.7	-33.9	-25.4	-22.8
**6**	DMSO : THF (2:1)	-59.6	-77.1	-53.0	-84.8	-31.3	-91.5	-97.7	-43.4	-24.4	-25.9	-25.9
**7**	DMSO : 1,2-dichlorobenzene (2:1)	-68.3	-79.0	-52.2	-79.6	-36.9	-87.1	-92.3	-52.6	-33.6	-26.8	-18.9
		-70.9	-72.9	-61.8	-80.6	-36.3		-93.3	-66.6			

Table S6 Selected TBC6-TBC6 structural motifs from the TBC6 solvates with 1,2,3-alternate conformation.

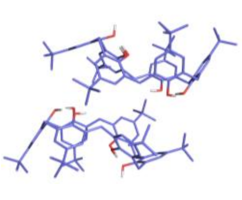

IV

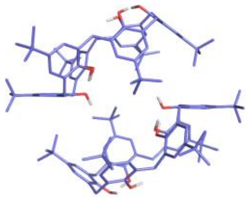

V

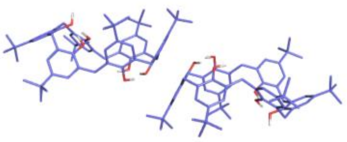

VI

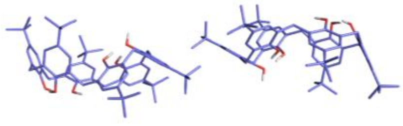

VII

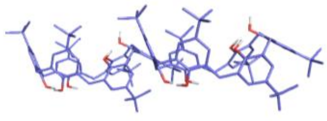

VIII

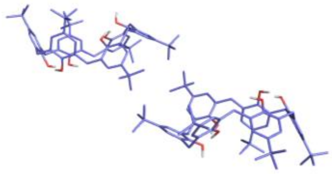

IX

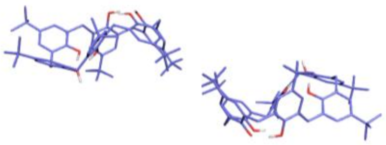

X

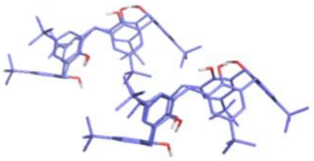

XI

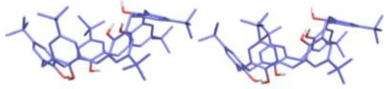

XII

Table S7 Energy frameworks of structures 1-7 along a, b, and c direction. Coulomb energy are shown in red, dispersion energy in green, and total energy in blue.

	a	*b*	*c*
	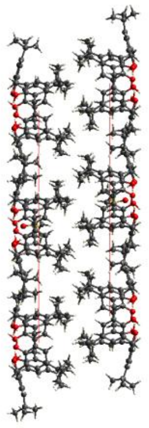	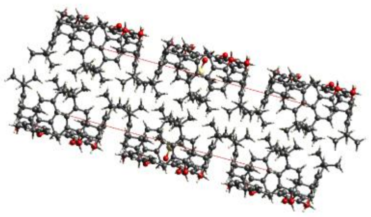	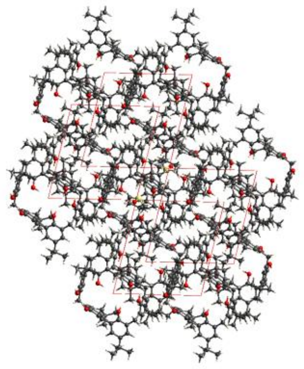
**1**	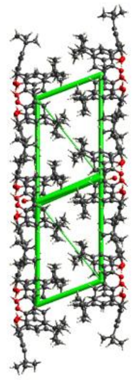	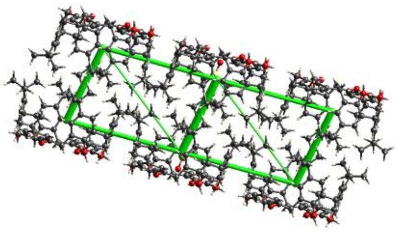	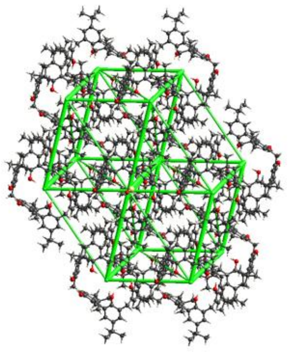
	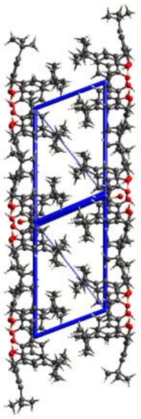	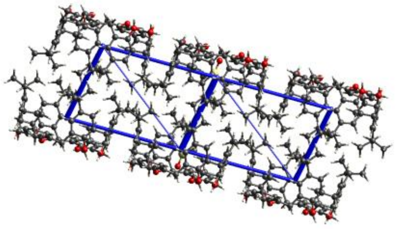	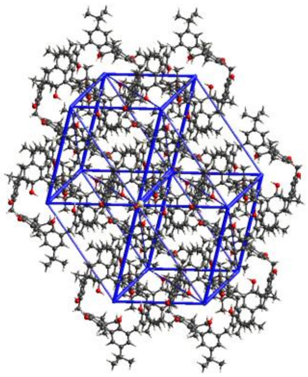

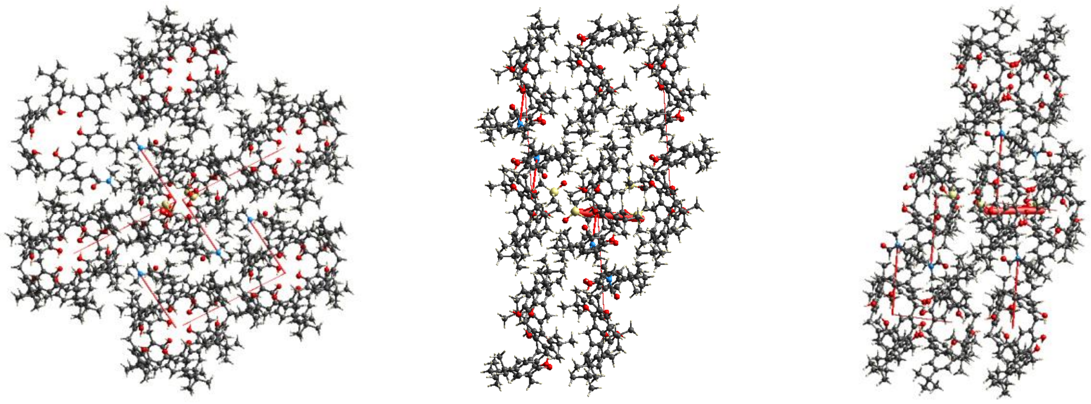

2

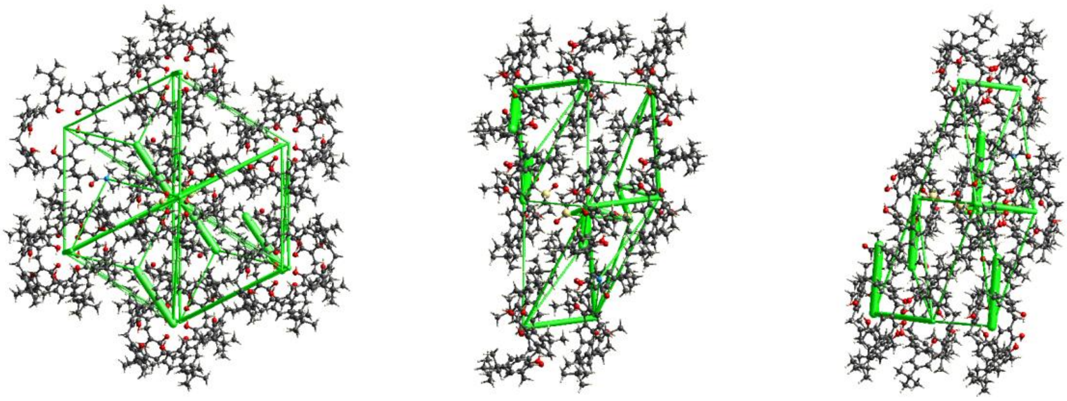

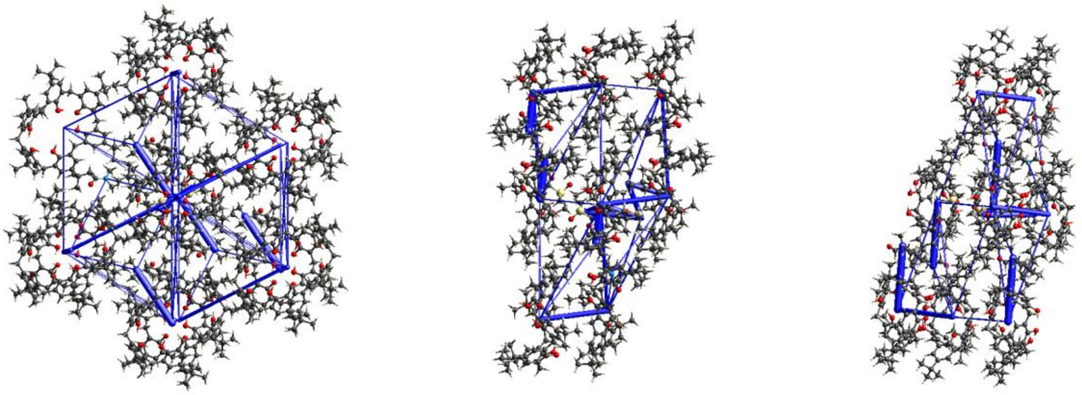

3

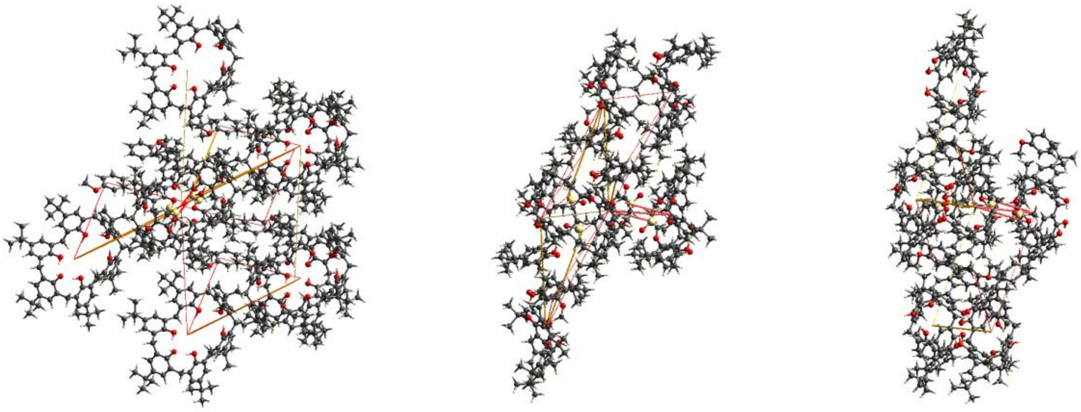

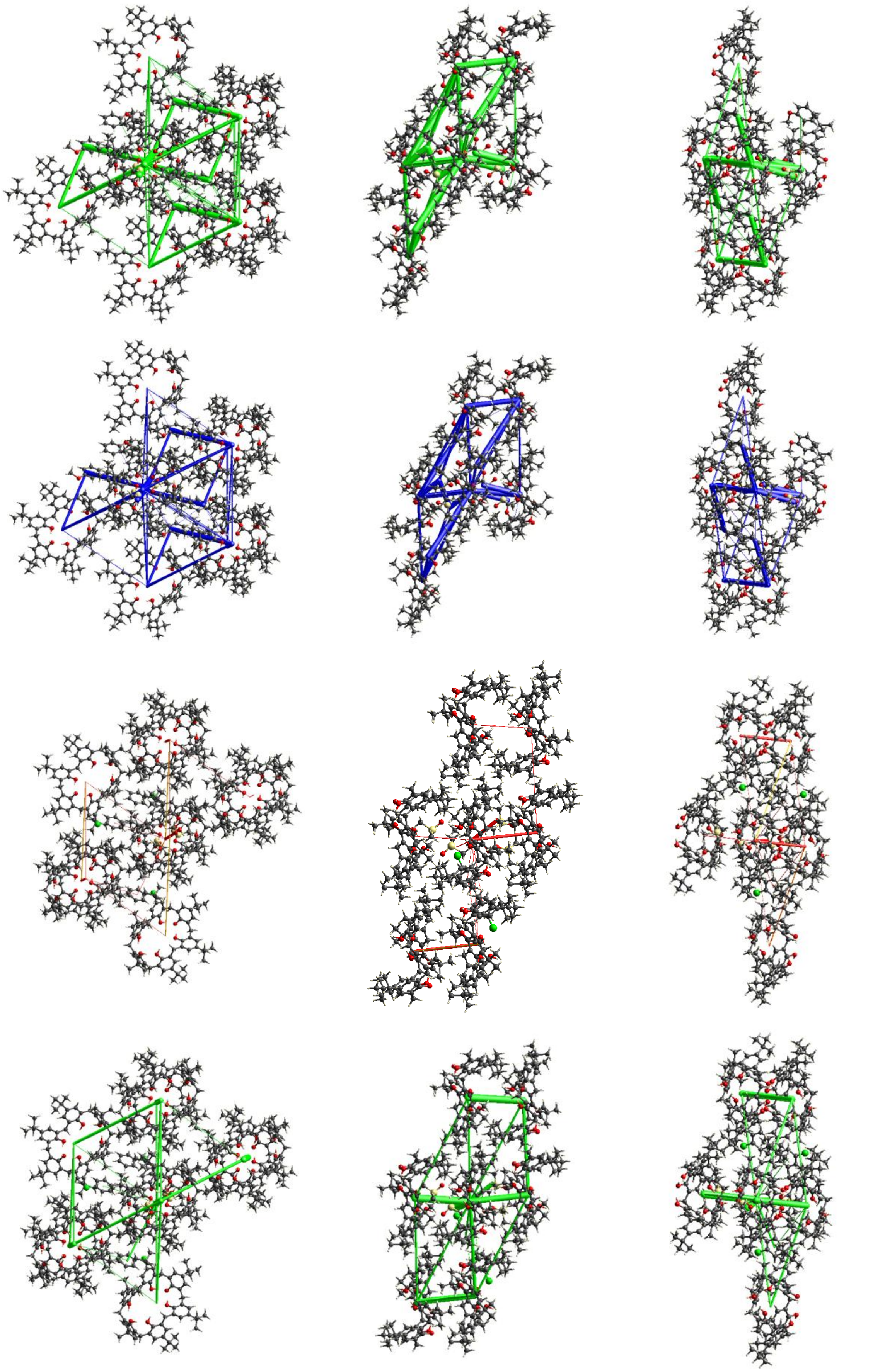

4

5

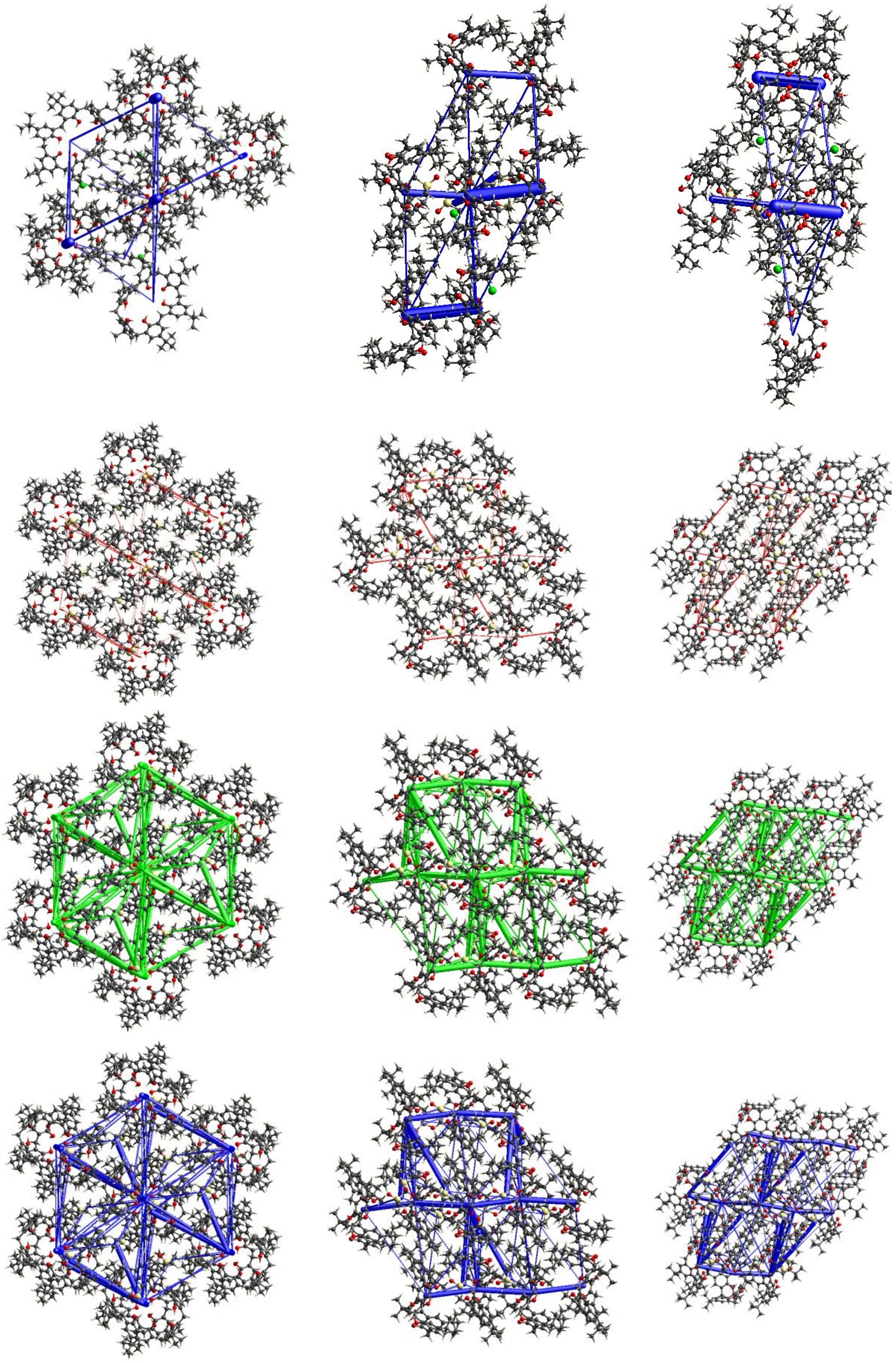

	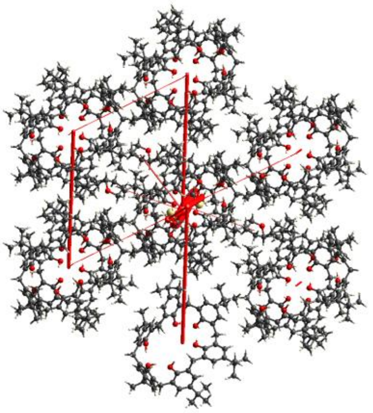	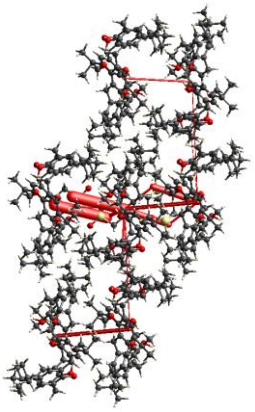	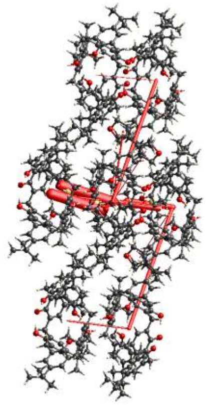
7	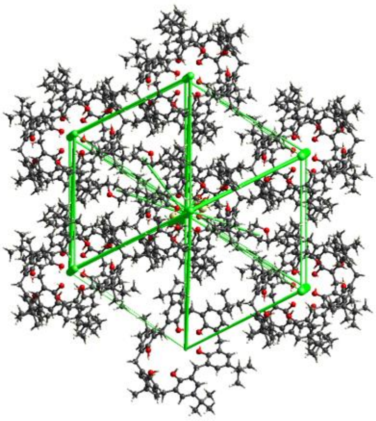	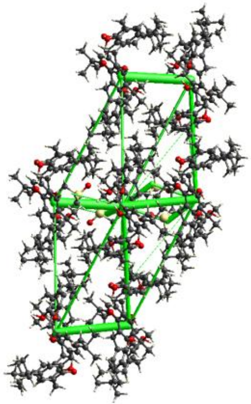	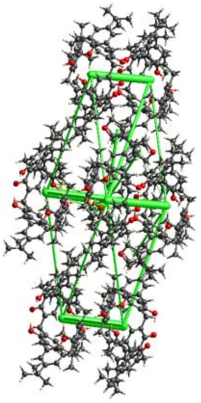
	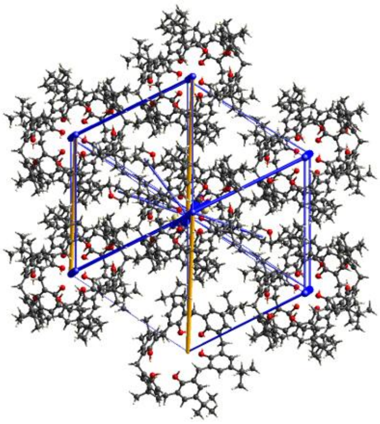	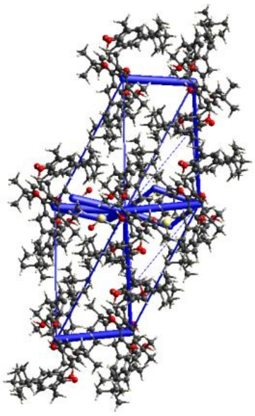	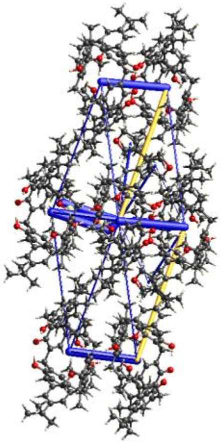
7	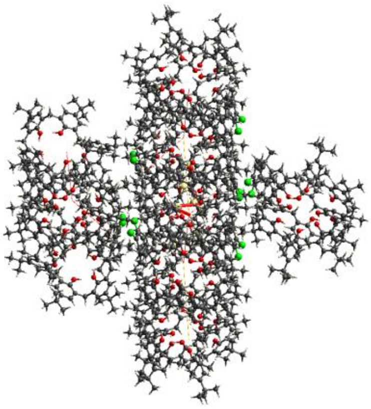	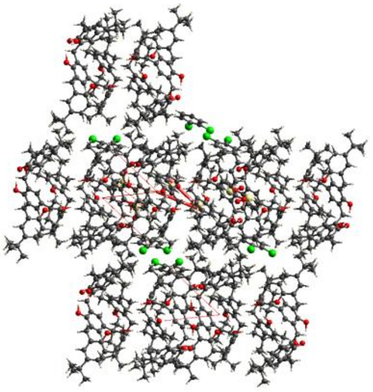	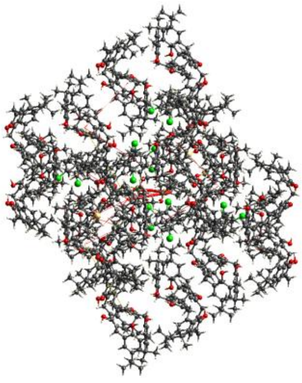

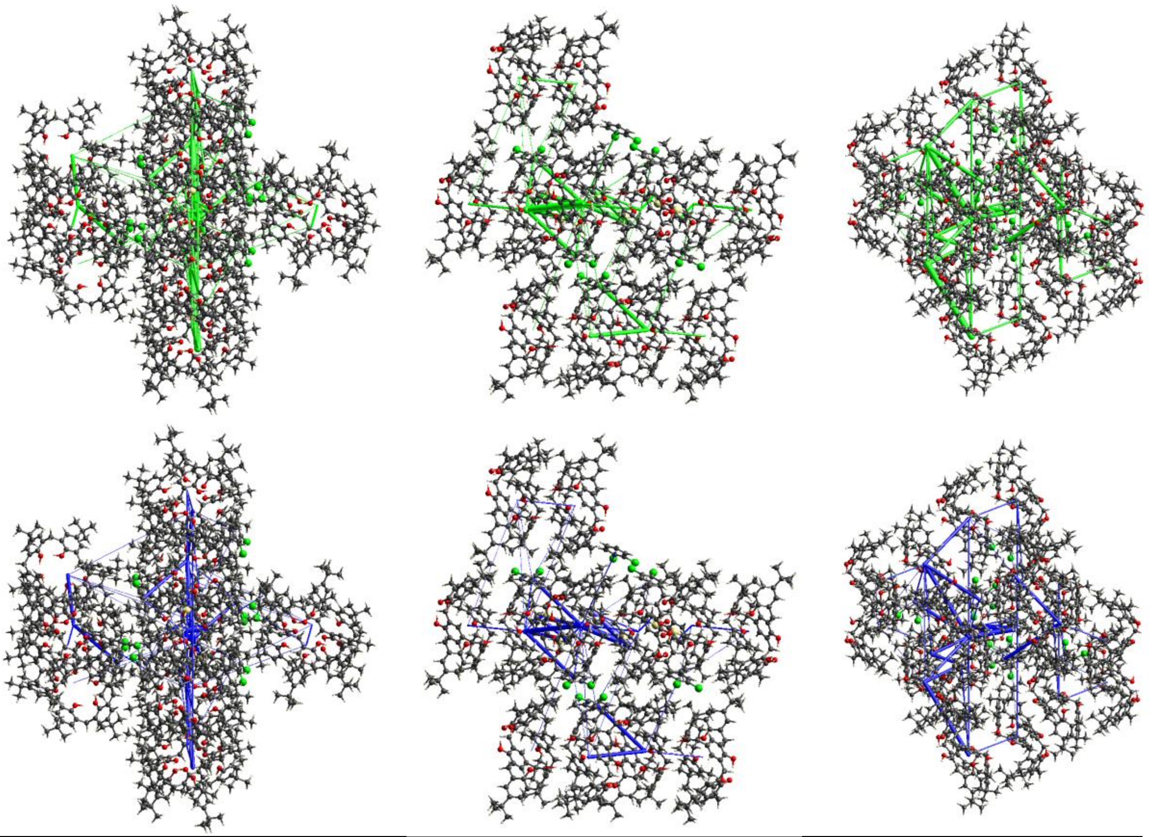

Table S8 Selected properties of solvents used for the solvent mixtures used in crystallization experiments.

	Density	Relative polarity	Dipole moment (D)	Dielectric constant	Molecular Weight	Topological Polar Surface Area [Å^2]	Standard enthalpy of formation $(\Delta_f H^{\ominus}_{298K})$ [kJ/mol]
DMSO	0.9445	0.386	3.86	36.71	73.09	20.3	-239.0
1,2-dichlorobenzene	1.3	0.19–0.20†	2.14	7.5	147	0	-17.4
1,3-chlorobenzene	1.288	0.17-0.18†	1.68	5	147	0	-20.5
1,4-chlorobenzene	1.283	0.11-0.12†	0	2.41	147	0	n/a
acetonitrile	0.810	0.586	1.70	17.5	74.1	20.2	-328.0
anisole	0.9956	0.198	1.36	4.45	108.14	9.2	-114.8
benzene	0.8765	0.111	0	2.28	78.11	0	-49
chlorobenzene	1.106	0.188	1.54	5.62	112.55	0	11.5
cyclohexane	0.779	0.006	0	1.88	84.16	0	-157.7
dichloromethan	0.791	0.762	1.60	33.0	32.0	20.2	-238.4
dimethylformamide	1.1	0.444	4.1	46.68	78.14	36.3	-203.4
ethanol	0.814	0.568	1.7	13.9	88.15	20.2	-351.0
ethyl acetate	0.807	0.228	n/a	10.4	102.2	20.2	-394.7
methyl acetate	0.927	0.253	1.69	6.7	74.08	26.3	-445.89

n-heptane	0.917	0.012	1.94	8.4	74.1	26.3	-430.5
pyridine	0.869	0.231	1.71	7.2	90.1	18.5	-379.0
tetrahydrofuran	0.805	0.327	2.76	18.51	72.11	17.1	-273.0
toluene	0.867	0.099	0.31	2.38	92.14	0	12

† Selected values of relative polarity are not a result of the experiments. These are estimated values based on the molecular structures.

Table S9 Summary of solvent properties observed in crystal structure 2-7.

Structure	Guest properties		
	Solvent	Solvent volume(Å^3)	Solvent volume in unit cell (%)
**1**	DMSO	1218.16	31.2
	DMSO	247.05	7.1
**2**	DMSO	268.76	7.7
	DMF	343.15	9.9
	Total	858.97	24.7
	DMSO	222.60	6.3
**3**	DMSO	244.23	7.0
	DMSO and anisole (0.66/0.34)	342.41	9.8
	Total	809.24	23.1
	DMSO	256.93	7.3
**4**	DMSO	242.05	6.8
	chlorobenzene and benzene (0.54/0.46)	371.67	10.5
	Total	870.65	24.6
	DMSO	284.61	8.2
**5**	DMSO	273.93	7.9
	DMSO	238.45	6.9
	Total	796.98	23.1
	DMSO	209.78	5.9
**6**	DMSO	225.20	6.4
	THF/1,3-dichlorobenzene (0.59/0.41)	340.02	9.6
	Total	775.00	22.0
	1,2-dichlorobenzene	983.32	6.8
	1,2-dichlorobenzene	938.10	6.5
	DMSO	713.05	5.0
**7**	DMSO	578.90	4.0
	DMSO	588.83	4.1
	DMSO	700.07	4.9
	Total	4502.27	31.3

Table S10 Selected hydrogen-bond parameters for structure **1**

D—H···*A*	*D*—H (Å)	H···*A* (Å)	*D*···*A* (Å)	*D*—H···*A* (°)
O1—H1···O13	0.84	1.96	2.754(11)	156.2
O3—H3···O2	0.84	1.88	2.702(11)	167.3
O4—H4···O13	0.84	2.05	2.831(11)	153.8
O6—H6···O5	0.84	1.82	2.661(12)	175.3
O7—H7···O15	0.84	2.07	2.692(18)	130.8
O7—H7···O14	0.84	2.23	2.77(3)	121.9
O9—H9···O8	0.84	1.96	2.719(11)	150.1
O10—H10···O14	0.84	1.96	2.75(3)	157.4
O11—H11···O12	0.84	1.84	2.667(11)	170.4

Table S11 Selected hydrogen-bond parameters for structure **2**.

D—H···*A*	*D*—H (Å)	H···*A* (Å)	*D*···*A* (Å)	*D*—H···*A* (°)
O2—H2···O1Si	0.84	1.68	2.517 (2)	171.5
O3—H3···O2	0.84	1.92	2.738 (2)	164.1
O5—H5···O6	0.84	1.92	2.742 (2)	166.0
O4—H4···O5	0.84	1.91	2.724 (2)	162.8
O6—H6···O2Sii	0.84	1.85	2.634 (2)	155.6
O1—H1···O2	0.84	1.90	2.721 (2)	163.9
C53—H53A···O7iii	0.98	2.04	2.86 (5)	140.4
C54—H54C···O7iii	0.98	2.05	2.87 (4)	140.3

Symmetry code(s): (i) -x+1, -y+1, -z+1; (ii) -x+2, -y+1, -z+1; (iii) -x+1, -y, -z+1.

Table S12 Selected hydrogen-bond parameters for structure **3.**

D—H···*A*	*D*—H (Å)	H···*A* (Å)	*D*···*A* (Å)	*D*—H···*A* (°)
O4—H4···O3	0.84	1.92	2.7378 (19)	163.5
O1—H1···O1S^{i}	0.84	1.67	2.509 (2)	172.2
O2—H2···O1	0.84	1.92	2.7322 (19)	163.2
O3—H3···O2S^{ii}	0.84	1.83	2.6214 (19)	155.4
O6—H6···O1	0.84	1.92	2.7318 (19)	162.9
O5—H5···O4	0.84	1.88	2.704 (2)	166.7

Symmetry code(s): (i) -*x*+1, -*y*+1, -*z*+1; (ii) -*x*, -*y*+1, -z+1.

Table S13 Selected hydrogen-bond parameters for structure **4**

D—H···*A*	*D*—H (Å)	H···*A* (Å)	*D*···*A* (Å)	*D*—H···*A* (°)
O3—H3···O2S	0.84	1.68	2.5184 (16)	172.4
O6—H6···O5	0.84	1.91	2.7341 (15)	168.0

O5—H5···O1S	0.84	1.83	2.6204 (15)	155.2
O4—H4···O3	0.84	1.93	2.7393 (15)	162.9
O2—H2···O3	0.84	1.91	2.7213 (15)	163.5
O1—H1···O6	0.84	1.90	2.7160 (16)	162.5

Table S14 Selected hydrogen-bond parameters for structure **5**

D—H···*A*	*D*—H (Å)	H···*A* (Å)	*D*···*A* (Å)	*D*—H···*A* (°)
O2—H2···O1	0.84	1.97	2.786 (2)	163.1
O5—H5···O2S	0.84	1.73	2.566 (2)	171.6
O3—H3···O2	0.84	1.90	2.732 (2)	169.7
O4—H4···O5	0.84	1.93	2.759 (2)	167.4
O1—H1···O1S	0.84	1.85	2.637 (2)	156.0

Table S15 Selected hydrogen-bond parameters for structure **6**

D—H···*A*	*D*—H (Å)	H···*A* (Å)	*D*···*A* (Å)	*D*—H···*A* (°)
O3—H3···O2S	0.84	1.67	2.508 (4)	173.6
O4—H4···O3	0.84	1.93	2.728 (4)	158.3
O2—H2···O3	0.84	1.93	2.728 (4)	157.2
O5—H5···O1S	0.84	1.83	2.618 (4)	155.1
O6—H6···O5	0.84	1.91	2.743 (4)	170.1
O1—H1···O6	0.84	1.87	2.699 (4)	166.7

Table S16 Selected hydrogen-bond parameters for structure **7**

D—H···*A*	*D*—H (Å)	H···*A* (Å)	*D*···*A* (Å)	*D*—H···*A* (°)
O1—H1···O1S	0.84	1.79	2.5960 (15)	159.4
O2—H2···O1	0.84	1.88	2.6975 (14)	163.3
O3—H3···O2	0.84	1.93	2.7553 (14)	165.2
O4—H4···O5	0.84	1.88	2.6856 (15)	160.0
O5—H5···O2S	0.84	1.72	2.5528 (15)	172.2
O6—H6···O5	0.84	2.00	2.8252 (15)	166.4
O7—H7···O3S	0.84	1.83	2.6176 (14)	155.3
O8—H8···O7	0.84	1.93	2.7531 (13)	166.8
O9—H9···O8	0.84	1.89	2.7202 (14)	168.5
O10—H10···O11	0.84	1.92	2.7379 (14)	164.6
O11—H11···O4S	0.84	1.70	2.5340 (15)	173.8
O12—H12A···O11	0.84	2.05	2.8536 (15)	160.7

a)

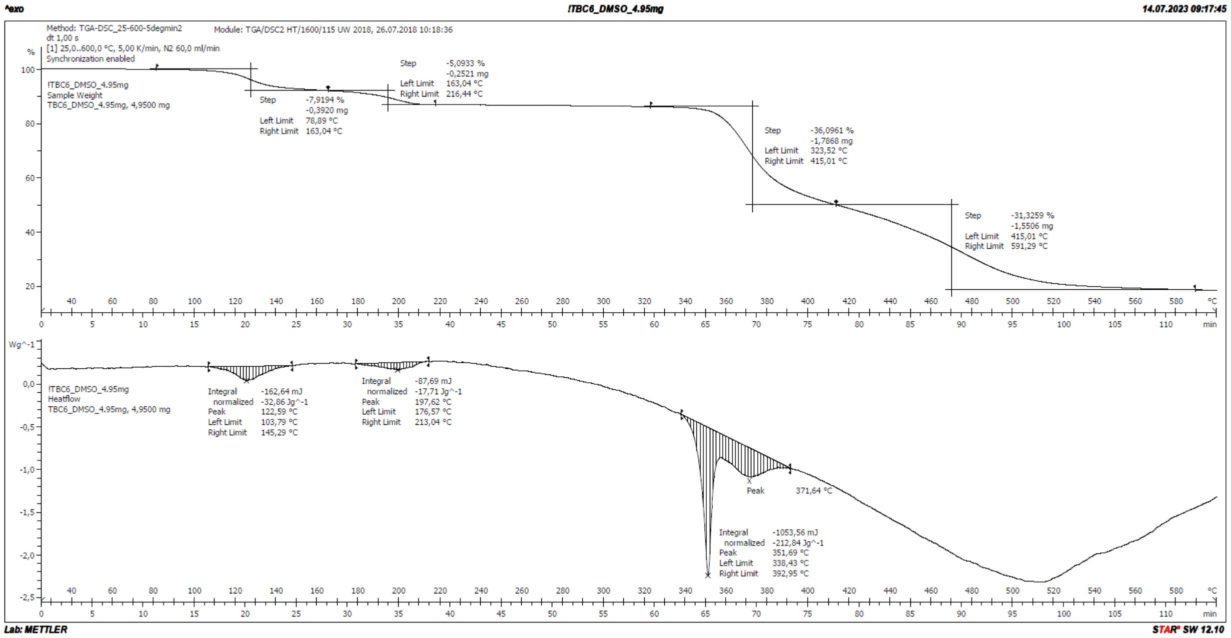

b)

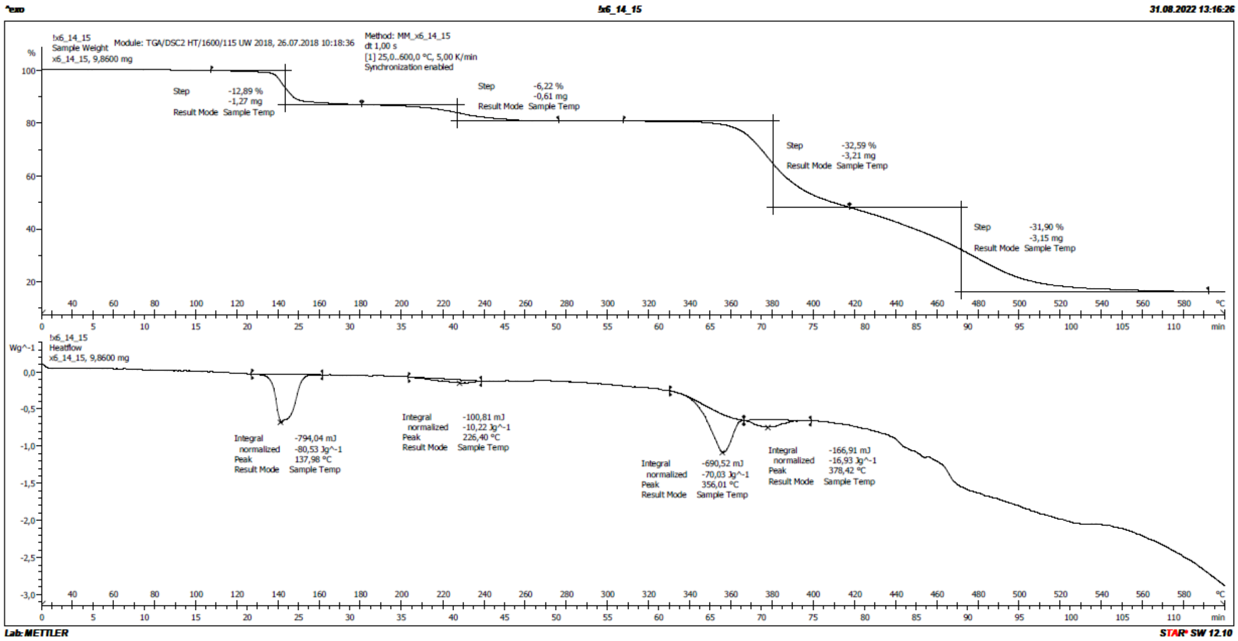

Figure S1 Results of TGA and DSC experiments of a) **5** and b) **2**.

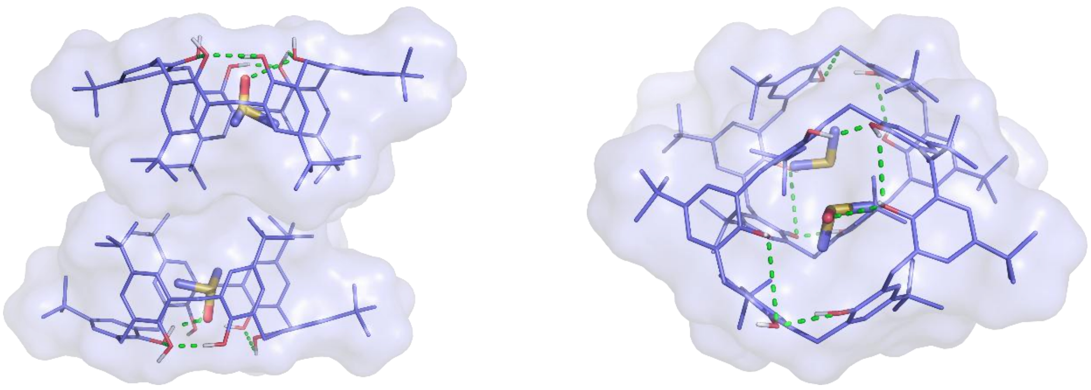

1

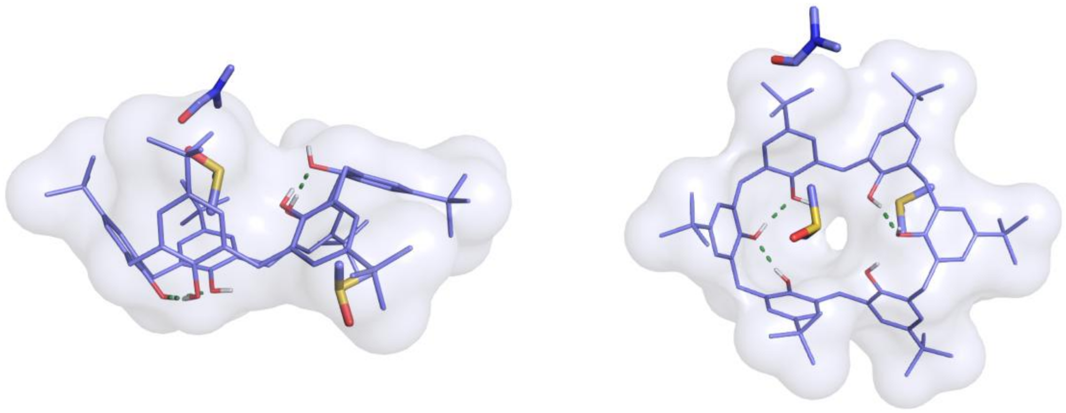

2

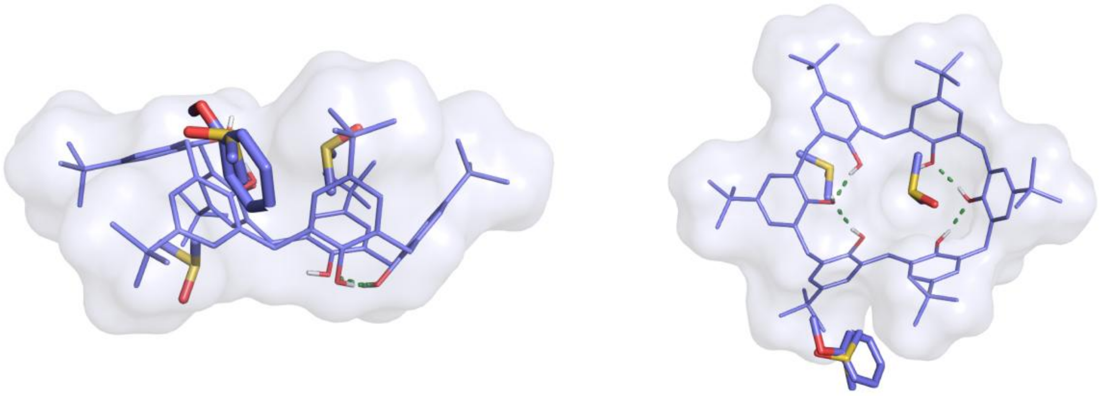

3

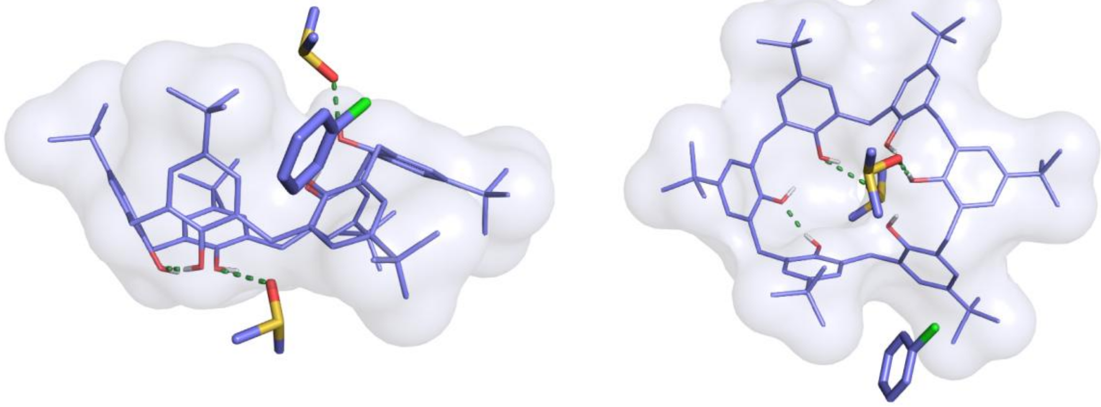

4

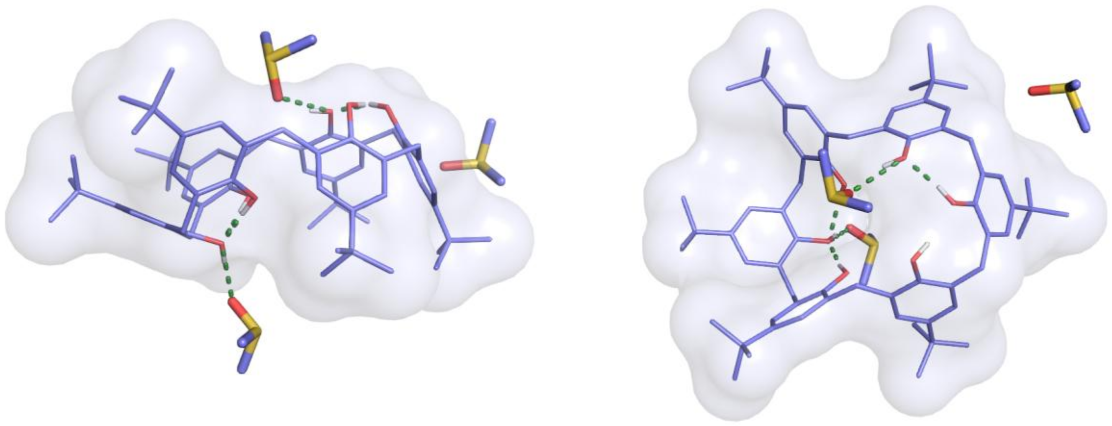

5

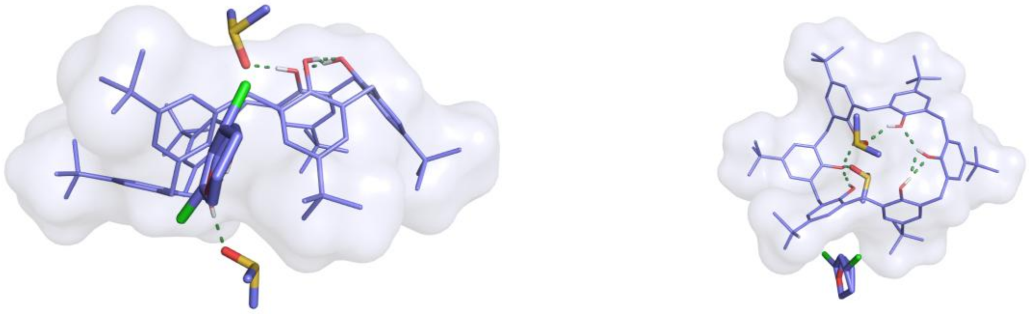

6

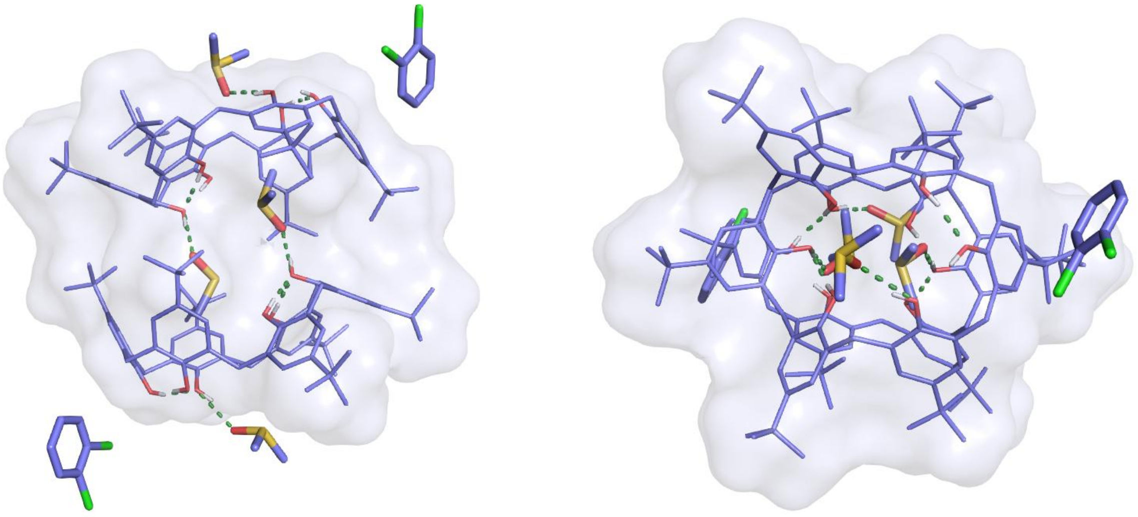

7

Figure S2 Asymmetric units of analysed solvates **1-7** presented with the view from the top and from side. Non-polar hydrogen atoms were omitted for clarity.

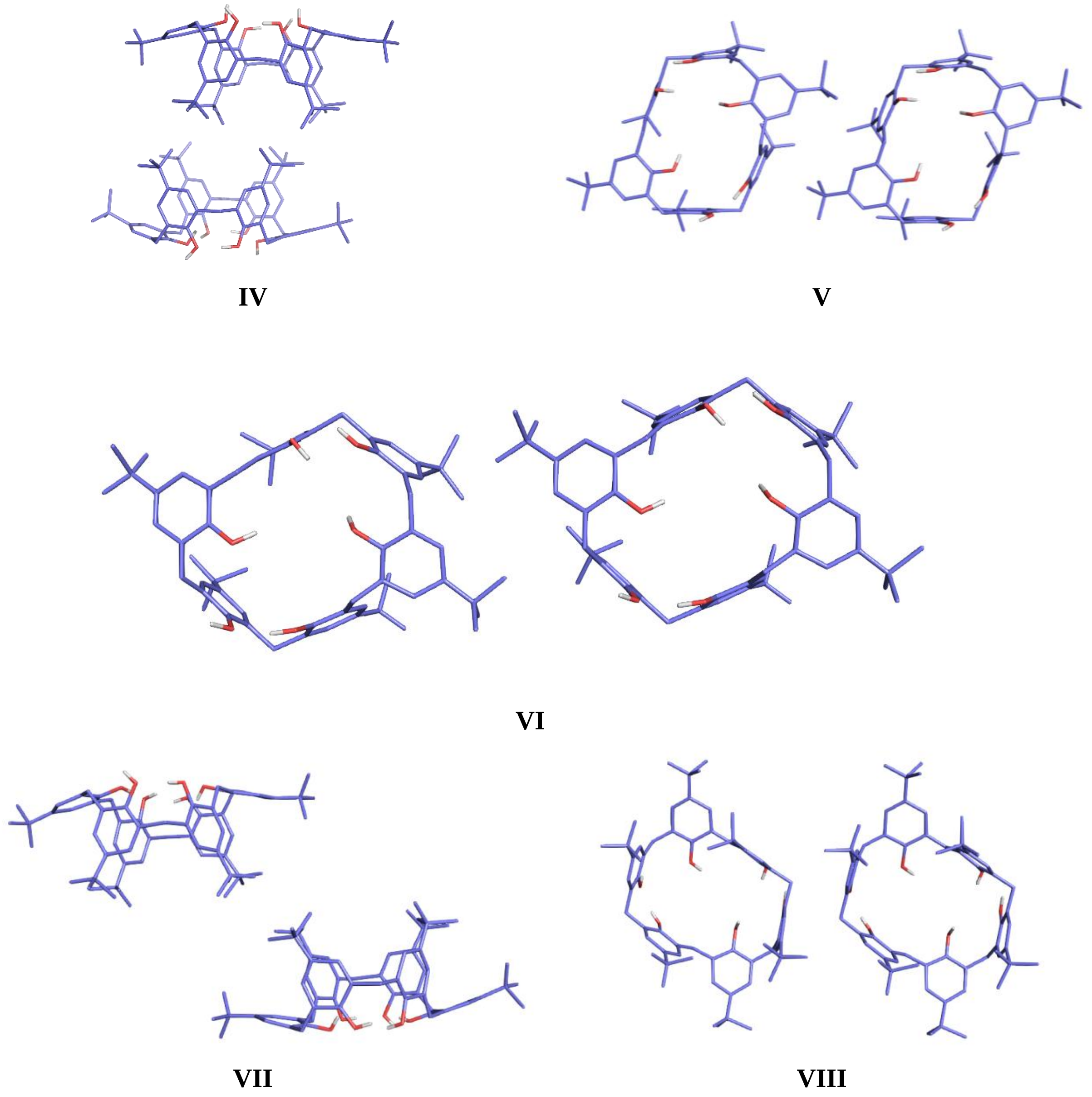

Figure S3 Selected TBC6-TBC6 structural motifs from the TBC6 solvates with winged cone conformation.

1

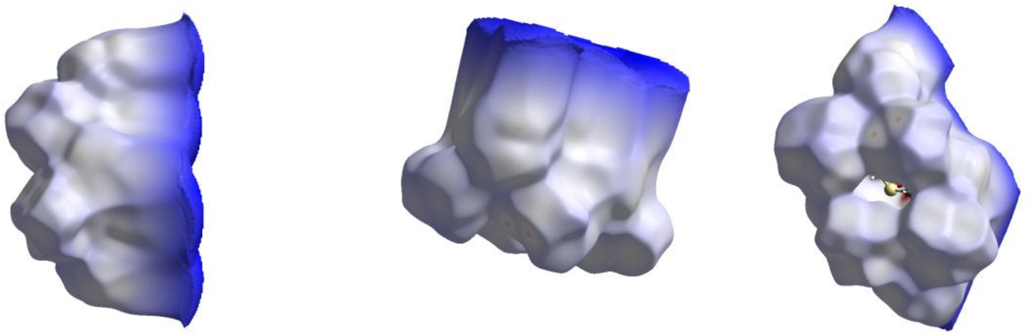

2

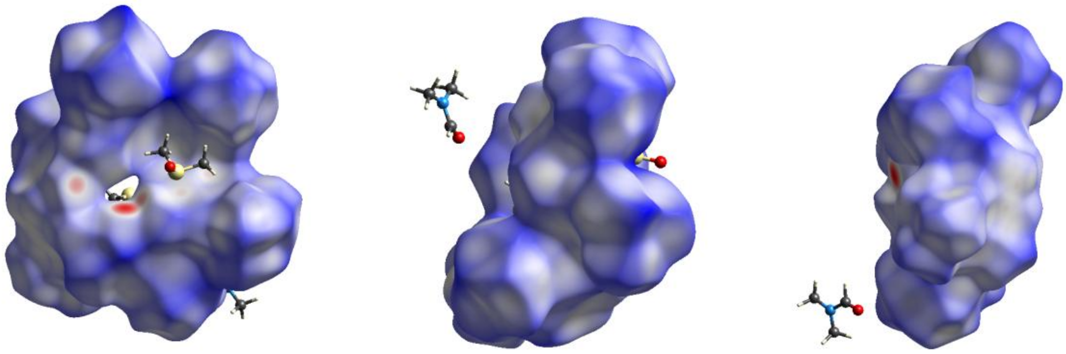

3

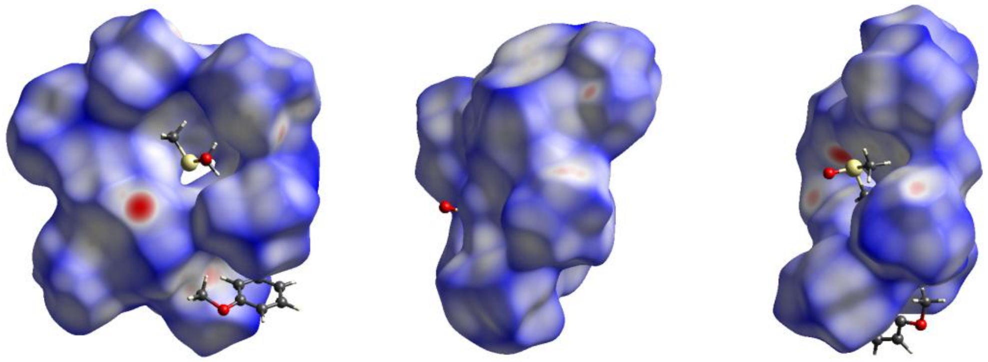

4

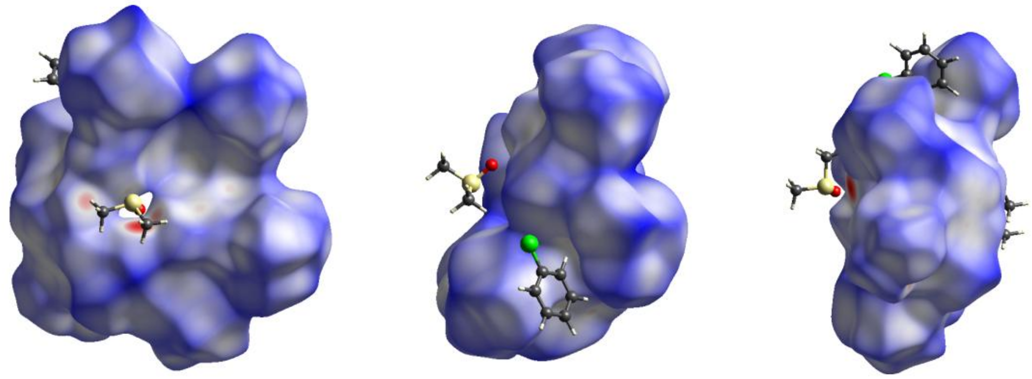

5

6

7

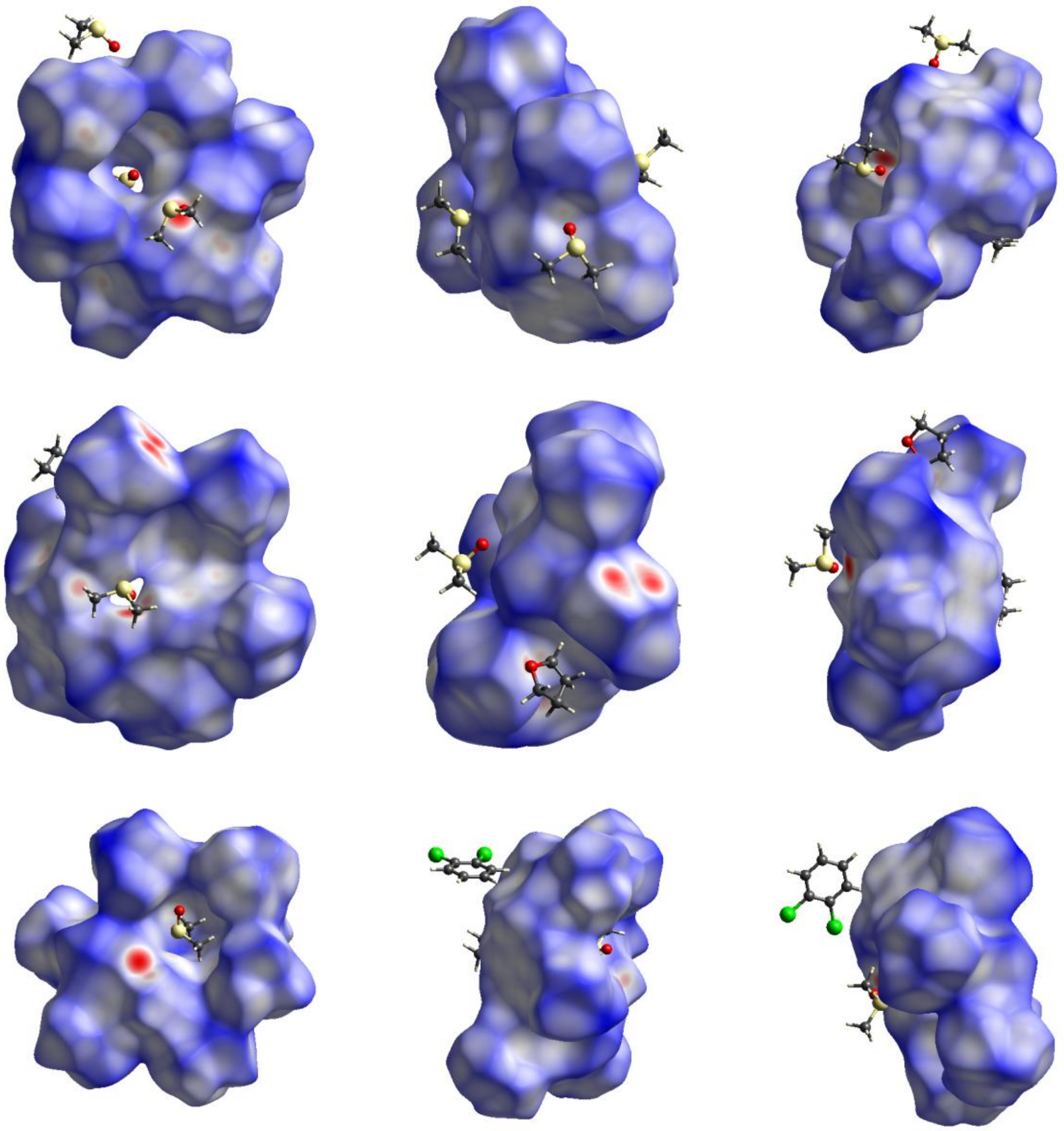

Figure S4 Hirshfeld surfaces of structures 1-7. Views in a, b, and c direction.
